# Peritoneal macrophage heterogeneity is associated with different peritoneal dialysis outcomes

**DOI:** 10.1016/j.kint.2016.10.030

**Published:** 2017-05

**Authors:** Chia-Te Liao, Robert Andrews, Leah E. Wallace, Mohd Wajid A. Khan, Ann Kift-Morgan, Nicholas Topley, Donald J. Fraser, Philip R. Taylor

**Affiliations:** 1 Systems Immunity University Research Institute and Division of Infection and Immunity, Cardiff University School of Medicine, Heath Park, Cardiff, UK; 2 Wales Kidney Research Unit, Cardiff University School of Medicine, Heath Park, Cardiff, UK

**Keywords:** dendritic cell, macrophage, peritoneal dialysis, peritoneal inflammation, peritonitis

## Abstract

Peritonitis remains the major obstacle for the maintenance of long-term peritoneal dialysis and dysregulated host peritoneal immune responses may compromise local anti-infectious defense, leading to treatment failure. Whilst, tissue mononuclear phagocytes, comprising macrophages and dendritic cells, are central to a host response to pathogens and the development of adaptive immune responses, they are poorly characterized in the human peritoneum. Combining flow cytometry with global transcriptome analysis, the phenotypic features and lineage identity of the major CD14^+^ macrophage and CD1c^+^ dendritic cell subsets in dialysis effluent were defined. Their functional specialization was reflected in cytokine generation, phagocytosis, and antigen processing/presentation. By analyzing acute bacterial peritonitis, stable (infection-free) and new-starter patients receiving peritoneal dialysis, we identified a skewed distribution of macrophage to dendritic cell subsets (increasing ratio) that associated with adverse peritonitis outcomes, history of multiple peritonitis episodes, and early catheter failure, respectively. Intriguingly, we also noted significant alterations of macrophage heterogeneity, indicative of different maturation and activation states that were associated with different peritoneal dialysis outcomes. Thus, our studies delineate peritoneal dendritic cells from macrophages within dialysate, and define cellular characteristics associated with peritoneal dialysis treatment failure. These are the first steps to unravelling the detrimental adaptive immune responses occurring as a consequence of peritonitis.

Peritoneal dialysis (PD)-related peritonitis remains a major cause of technique failure as well as mortality in maintenance PD patients.^1^, ^2^ In PD patients, the dialysis exchange perturbs the tightly regulated tissue homeostasis and composition of the immune cell population within the peritoneal cavity.^3^ Previously, we have delineated the local response to peritoneal infection–driven inflammation.^4^, ^5^ Our recent work has shown that repeated bacterial peritoneal inflammation drives immune-mediated compromised tissue repair response and fibrosis.^6^ These data support the concept that alterations in peritoneal immune cell composition in PD patients may link to their adverse outcomes.

Tissue mononuclear phagocytes, comprised mainly of macrophages (MØ) and dendritic cells (DC), are a key tissue-resident component of the local immune system, with roles including pathogen clearance, tissue repair, and antigen presentation.^7^, ^8^ Recent progresses in MØ/DC biology have uncovered heterogeneity in these cell phenotypes, related to different developmental origins and distinct differentiation pathways.^9^, ^10^, ^11^ Studies on the phenotypic and functional attributes of different tissue MØ/DC subsets *in vivo* have highlighted that distinct subsets have specialized roles in tissue homeostasis and local inflammation.^12^, ^13^ In the peritoneal cavity, studies have been largely focused on MØ in mice. We, and others, have recently identified a major, self-renewing population of tissue-resident macrophages, with roles in tissue homeostasis and response to inflammation.^14^, ^15^, ^16^ On acute peritoneal inflammation induced by *Staphylococcus epidermidis* supernatant in mice,^17^ neutrophils and monocytes are recruited into the inflamed peritoneum. These infiltrated monocytes will differentiate into MØ and/or DC and play effector functions locally (i.e., phagocytosis and apoptotic cell clearance, antigen presentation, and T-cell stimulation). In the field of PD, studies in late 1970s began to examine the cellular composition of dialysis effluent fluids from noninfected PD patients, and revealed the MØ as the predominant cell type found in dialysis effluent.^18^, ^19^ Based on *ex vivo* and *in vitro* functional analysis, peritoneal MØs are important in the front line of host peritoneal defense in PD patients.^20^, ^21^, ^22^, ^23^ It has been suggested that peritoneal MØs from PD patients phenotypically and functionally resemble *in vitro* polarized macrophage colony-stimulating factor–driven “anti-inflammatory” MØ or interleukin (IL)-4–driven alternatively activated MØ^24^, ^25^; however, so far the comprehensive genetic profiling and related biological functional pathways of peritoneal MØ from PD effluents remains lacking and these conclusions overlook the likely complex cell-cell heterogeneity. In contrast with the study of peritoneal MØs, information on the DC component of the human peritoneal cavity is sparse, especially in the context of PD. Historically this fundamental knowledge-deficiency is attributed to the paucity of specific markers needed for the unambiguous identification of this cell type.^26^ Overall, the lack of detailed phenotypic and functional characterization of human peritoneal mononuclear phagocytes has limited the investigation of their specialized roles in the context of PD-related peritonitis, immune dysfunction, and tissue damage.

To address this and to understand how the patient’s peritoneal immune system responds to pathogen invasion, we first aimed to define peritoneal mononuclear phagocyte subsets, phenotypically and functionally during stable PD and in patients experiencing peritonitis. Second, we longitudinally monitored the kinetic changes of peritoneal MØ/DC subsets in the new-starter PD patients to explore the patient’s immune response to the catheter insertion surgery and dialysis exchange.

We established novel criteria for the specific identification of distinct peritoneal MØ/DC subsets in PD patients. This has permitted a comprehensive analysis of their heterogeneity and we have defined the immunological differences between the cell types by global transcriptome analysis and specific functional assessment. Finally, we demonstrate that there are alterations of phenotypic distribution of peritoneal mononuclear phagocyte subsets under defined clinical settings, which are associated with patient outcomes.

## Results

### Phenotypic identification of 2 distinct peritoneal mononuclear phagocyte subsets

**Figure 1:**
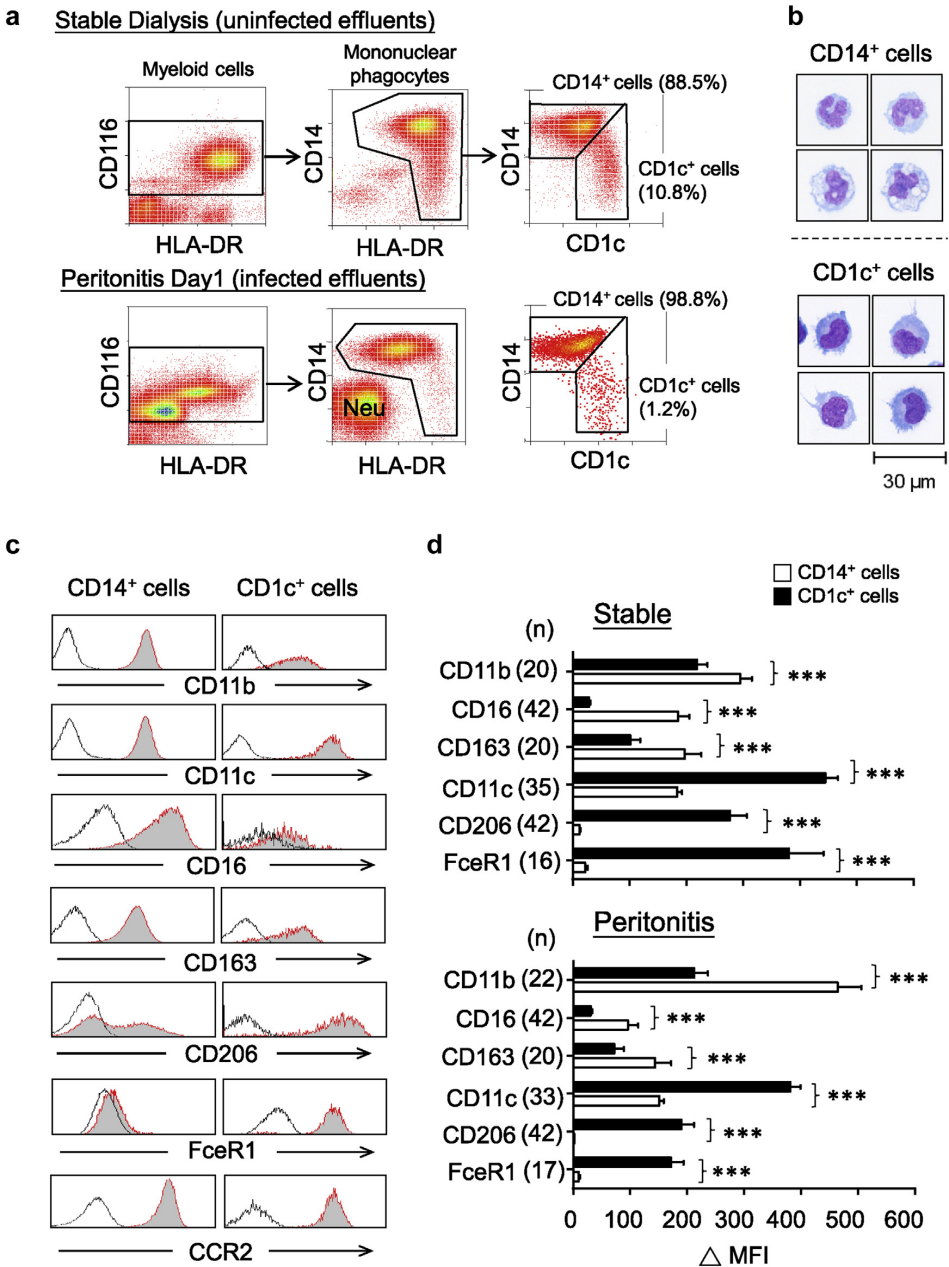
**Phenotypic identification of peritoneal mononuclear phagocyte subsets.** (**a**) Representative density plots showing flow-cytometric gating strategies to identify peritoneal mononuclear phagocyte subsets within PD effluent from stable dialysis patients (upper panel) and day 1 peritonitis patients (lower panel). Myeloid cells were pregated on CD116^+^ populations after exclusion of doublets, cellular debris, and dead cells. Within these cells, mononuclear phagocytes could be readily identified as HLA-DR^+^CD14^+/‒^, and granulocytes were HLA-DR^‒^CD14^‒^. The latter mainly comprised CD16^high^ neutrophils (Neu), which substantially increased in number during acute peritonitis. Mononuclear phagocytes could be segregated into 2 subsets: CD14^+^CD1c^low/‒^ (major) and CD1c^+^CD14^low/‒^ (minor). (**b**) Sorted CD14^+^ cells and CD1c^+^ cells (the purity >95%) were cytospun, air-dried, and stained with Microscopy Hemacolor. The morphology of cells is shown (bar = 30 μm). Data derived from 1 patient representative of 4 stable patients. (**c**) Flow-cytometric analysis of select marker expression by CD14^+^ cells and CD1c^+^ cells. Representative histogram plots are pregated as described previously. Shaded histograms depict receptor-specific staining and bold lines denote isotype control staining. Data are derived from 1 patient representative of 42 stable patients giving similar results. (**d**) Bar graphs shown were quantification of receptor expression, measured as difference in medium fluorescence intensity (MFI) between receptor-specific and isotype control staining. Quantitative comparisons were made between CD14^+^ cells and CD1c^+^ cells under stable status (upper) and during peritonitis (lower). Data are shown as mean ± SEM of indicated number of patients (*n*) and were analyzed using paired *t* test.

Although detailed analysis of the composition of leukocyte subpopulations within the PD effluents has improved,^27^ the phenotypic characterization of mononuclear phagocytes is still relatively poor. Here, we used a novel flow-cytometric gating strategy based on CD116 (granulocyte-macrophage colony-stimulating factor receptor alpha) expression (Figure 1a), a “pan-myeloid lineage” cell marker, which has been used effectively in mice.^28^ Together with human leukocyte antigen–antigen D related (HLA-DR) (major histocompatibility complex class II molecule) and CD14 (a coreceptor for lipopolysaccharide), CD116^+^ myeloid cells could be separated into HLA-DR^+^CD14^+/‒^ mononuclear phagocytes and HLA-DR^‒^CD14^‒^ granulocytes. The latter, discussed in more detail later in this article, mainly comprised CD16^high^ neutrophils that substantially increased in number during acute bacterial peritonitis (Figure 1a). Notably, 2 distinctive mononuclear phagocyte subsets could be recognized by the additional staining of CD1c antigen (blood dendritic cell antigen 1):CD14^+^CD1c^low/‒^ and CD1c^+^CD14^low/‒^. Both subsets were morphologically monocytic in appearance, but CD1c^+^ cells were smaller in size with less cytoplasm (Figure 1b). Phenotypically, CD14^+^ cells displayed higher CD11b, CD16 (FcγRIII), and CD163 expression, whereas CD1c^+^ cells had higher CD11c, FcɛR1α, and CD206 (mannose receptor) expression; both subsets are CCR2^+^ (Figure 1c). Examination of the dialysate of PD patients with acute bacterial peritonitis revealed similar marker expression when compared with stable patients without peritonitis (Figure 1d).

### Transcriptional profiling delineates the identities of mononuclear phagocyte subsets

**Figure 2:**
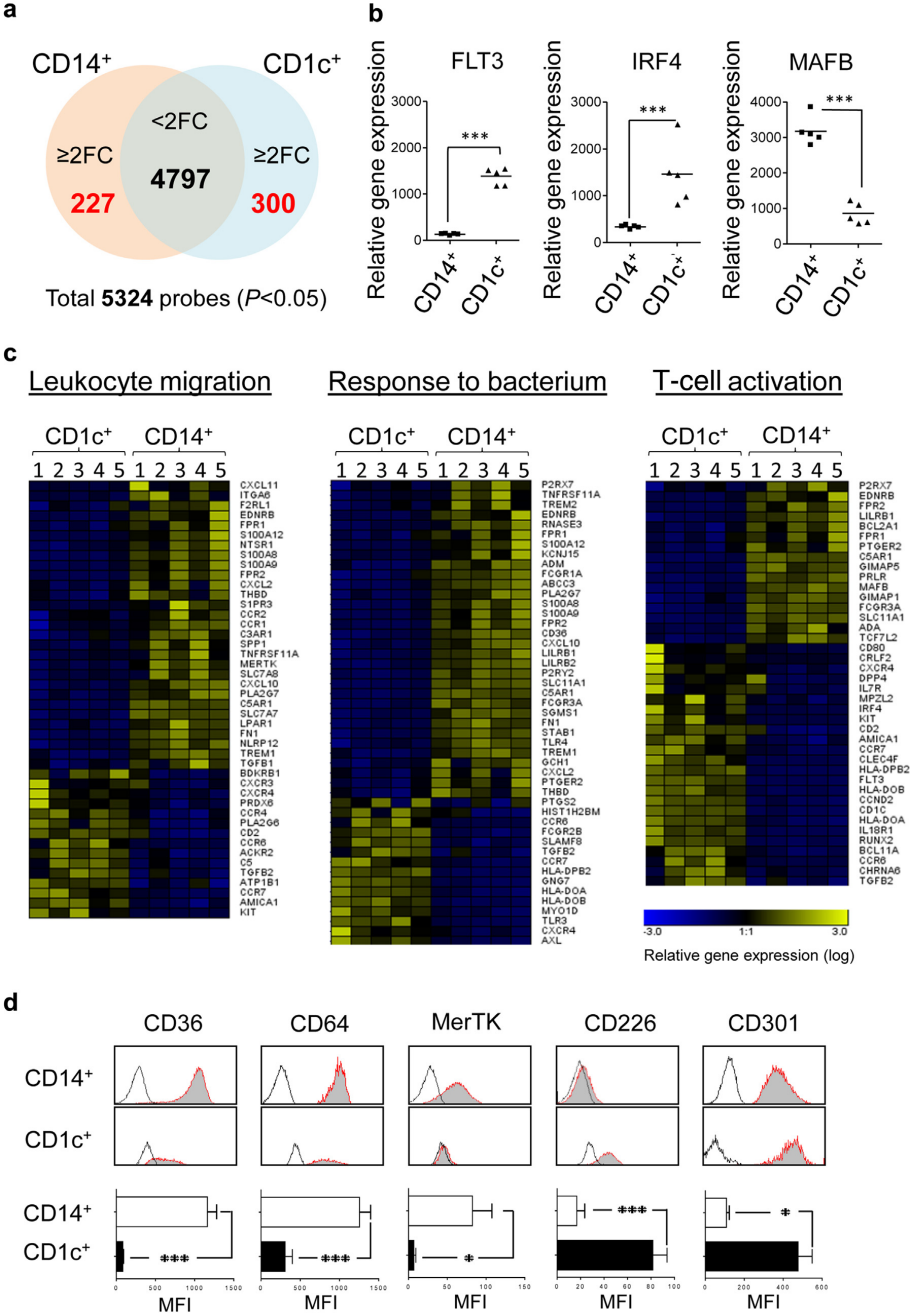
**Transcriptional profiling of 2 distinct peritoneal mononuclear phagocytes.** Gene expression profiles of purified peritoneal CD14^+^ cells and CD1c^+^ cells within PD effluent from 5 stable dialysis patients were analyzed by Affymetrix microarrays (Affymetrix GeneChip Human Gene 1.0 ST Array). (**a**) Venn diagram showing the number of the differentially expressed gene probesets identified via microarray analysis (filtered by adjusted *P* < 0.05). Among 5324 identified gene probesets, 4797 gene probesets are identified as less than 2 fold changes between CD14^+^ cells and CD1c^+^ cells. There are 227 gene probesets upregulated ≥2 fold changes on CD14^+^ cells (compared with CD1c^+^ cells), whereas 300 gene probesets upregulated ≥2 fold changes on CD1c^+^ cells (compared with CD14^+^ cells). (**b**) Expression of mRNA encoding FLT3, IRF4 (both for “DC development”) and MAFB (“MØ development”) indicated by the Affymetrix analysis. (**c**) Heat maps representing the relative gene expression of immune functional pathways, including “leukocyte migration,” “response to bacteria,” and “T-cell stimulation.” Differentially expressed genes selected for analysis were based on fold change ≥2, either CD14^+^ cells versus CD1c^+^ cells or vice versa, and then clustered on heat maps. (**d**) Flow-cytometric analysis of select marker expression by CD14^+^ MØs and CD1c^+^ DCs. Representative histogram plots are pregated as described in Figure 1. (Upper panels) Shaded histograms depict receptor-specific staining and bold lines denote isotype control staining. Data are derived from 1 patient representative of 7 stable patients. (Lower panels) Bar graphs shown were quantification of receptor expression, measured as the difference in medium fluorescence intensity (MFI) between receptor-specific and isotype control staining. Quantitative comparisons were made between CD14^+^ MØs and CD1c^+^ DCs from stable dialysis patients (*n* = 7). Data are shown as mean ± SEM and were analyzed using paired *t* test.

To determine how distinct the CD1c^+^ cells were, we analyzed the transcriptome of the CD14^+^ and CD1c^+^ subsets purified from uninfected PD effluent of stable patients (*n* = 5). Microarray data identified a unique transcriptional profile of differentially expressed genes in each cell subset (Figure 2a). Notably, the CD1c^+^ subset displayed significantly higher *FLT3* (Fms-related tyrosine kinase 3) and *IRF4* (Interferon Regulatory Factor 4) expression, both of which are involved in DC development,^29^, ^30^ whereas the CD14^+^ subset had higher *MAFB* (V-maf musculoaponeurotic fibrosarcoma oncogene homolog B) expression, which is associated with MØ differentiation (Figure 2b).^31^ Canonical pathway analysis further revealed differentially expressed genes in CD1c^+^ cells were more involved in DC maturation and antigen presentation, whereas CD14^+^ cells were enriched in differentially expressed genes associated with cholesterol biosynthesis, eicosanoid signaling, and the complement system (Supplementary Figure S1). Together, these data implied that the CD1c^+^ subset could be the “*bona-fide* DC” subset in the human peritoneal cavity, whereas the CD14^+^ subset had a transcriptional profile characteristic of “traditional MØs.” Furthermore, CD14^+^ MØs and CD1c^+^ DCs exhibited distinctive expression profiles in a number of biological processes, as exemplified by those involving in leukocyte migration, responses to bacteria, and T-cell stimulation (Figure 2c). Additionally, there were evident differences in genes involved in pathogen recognition (Toll-like receptors, c-type lectin receptors), receptor-mediated endocytosis/phagocytosis (scavenger receptors, Fc receptors), chemokine or cytokine system, and antigen presentation (Supplementary Figure S2). Of note, CD14^+^ MØs had higher *CD36*, *CD64*, *MerTK* (MER proto-oncogene, tyrosine kinase) expression, whereas CD1c^+^ DCs displayed higher *CD226* and *CLEC10A (CD301)* expression; and these were validated by flow cytometry through specific staining of these surface molecules (Figure 2d). Interestingly, recent studies show that these markers are useful for phenotyping multiple tissue MØ/DC subsets in mice.^17^, ^32^

### Peritoneal DC maturation during bacterial peritonitis

**Figure 3:**
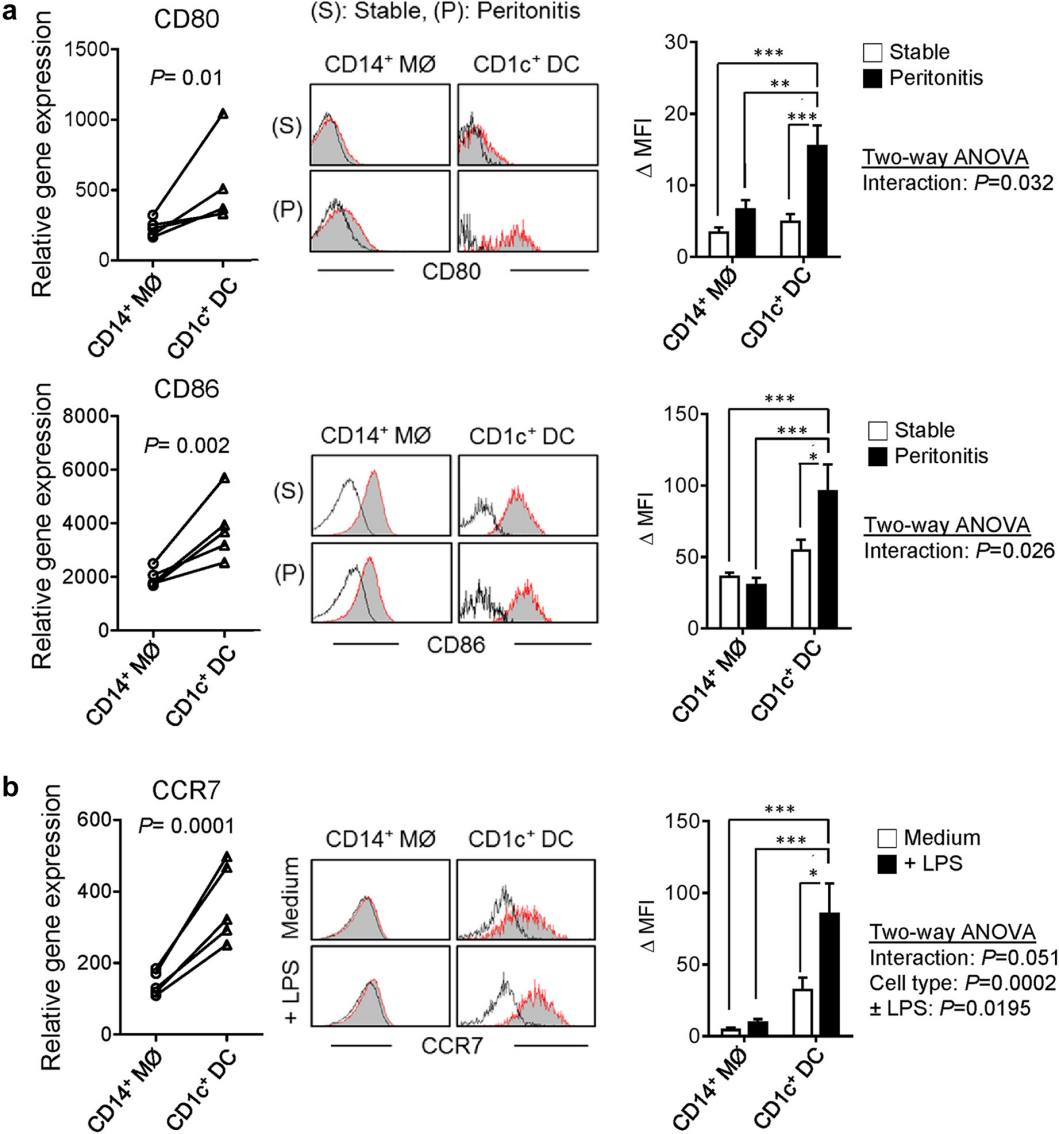
**Peritoneal dendritic cell maturation on peritoneal infections.** (**a**) (Left graphs) Microarray analysis showing differential expression of *CD80* and *CD86* genes between CD14^+^ MØ and CD1c^+^ DC. Data derived from 5 individual stable patients were analyzed by *t* test with multiple adjustment. (Middle panels) Representative histogram plots depicting flow-cytometric analysis of surface expression of CD80 and CD86 on CD14^+^ MØ and CD1c^+^ DC, respectively, from stable dialysis and peritonitis (day 1) patients. Data represent 1 of a total 20 stable patients and 1 of a total 20 peritonitis patients. (Right graphs) Quantification of CD80 and CD86 expression, measured as difference in medium fluorescence intensity (MFI) between receptor-specific and isotype control staining. Quantitative comparisons were made between CD14^+^ cells and CD1c^+^ cells under stable status (*n* = 20) and during peritonitis (*n* = 20). Data are shown as mean ± SEM and were analyzed using 2-way analysis of variance (ANOVA) with the Tukey multiple comparisons test. (**b**) (Left graph) Microarray analysis showing differential expression of *CCR7* gene between CD14^+^ MØ and CD1c^+^ DC. Data derived from 5 individual stable patients were analyzed by *t* test with multiple adjustment. (Middle panels) Representative histogram plots depicting flow-cytometric analysis of surface expression of CCR7 on CD14^+^ MØ and CD1c^+^ DC, respectively, with and without *ex vivo* lipopolysaccharide stimulation for 24 hours. Data represent 1 of 7 stable patients. (Right graph) Quantitative comparisons of CCR7 expression (ΔMFI) were made between CD14^+^ cells and CD1c^+^ cells with and without *ex vivo* lipopolysaccharide stimulation (*n* = 7). Data are shown as mean ± SEM and were analyzed using 2-way analysis of variance (ANOVA) with the Tukey multiple comparisons test. ns, *P* ≥ 0.05; *P < 0.05; **P < 0.01; ***P < 0.001.

Upregulation of costimulatory molecules, CD80 and CD86, on mature DCs is important for antigen presentation and T-cell activation. Our transcriptomic data revealed that CD1c^+^ DCs express higher levels of *CD80* and *CD86* than CD14^+^ MØs (Figure 3a, left panels). Notably, significantly increased expression of CD80 and CD86 expression in CD1c^+^ DCs was observed during acute bacterial peritonitis, compared with stable status (Figure 3a, middle and right panels). Additionally, peritoneal DCs exhibited higher *CCR7* (C-C motif chemokine receptor 7) expression than MØs (Figure 3b, left panel). CCR7 is important for tissue DC migration to draining lymph nodes, where mature DCs could activate T cells to initiate their effector functions.^33^ Importantly, considerable upregulation of CCR7 in DCs was observed on *ex vivo* lipopolysaccharide stimulation, implying that some DCs would acquire migratory properties during maturation (Figure 3b, middle and right panels).

### Characteristic functional specialization of peritoneal MØ and DC

**Figure 4:**
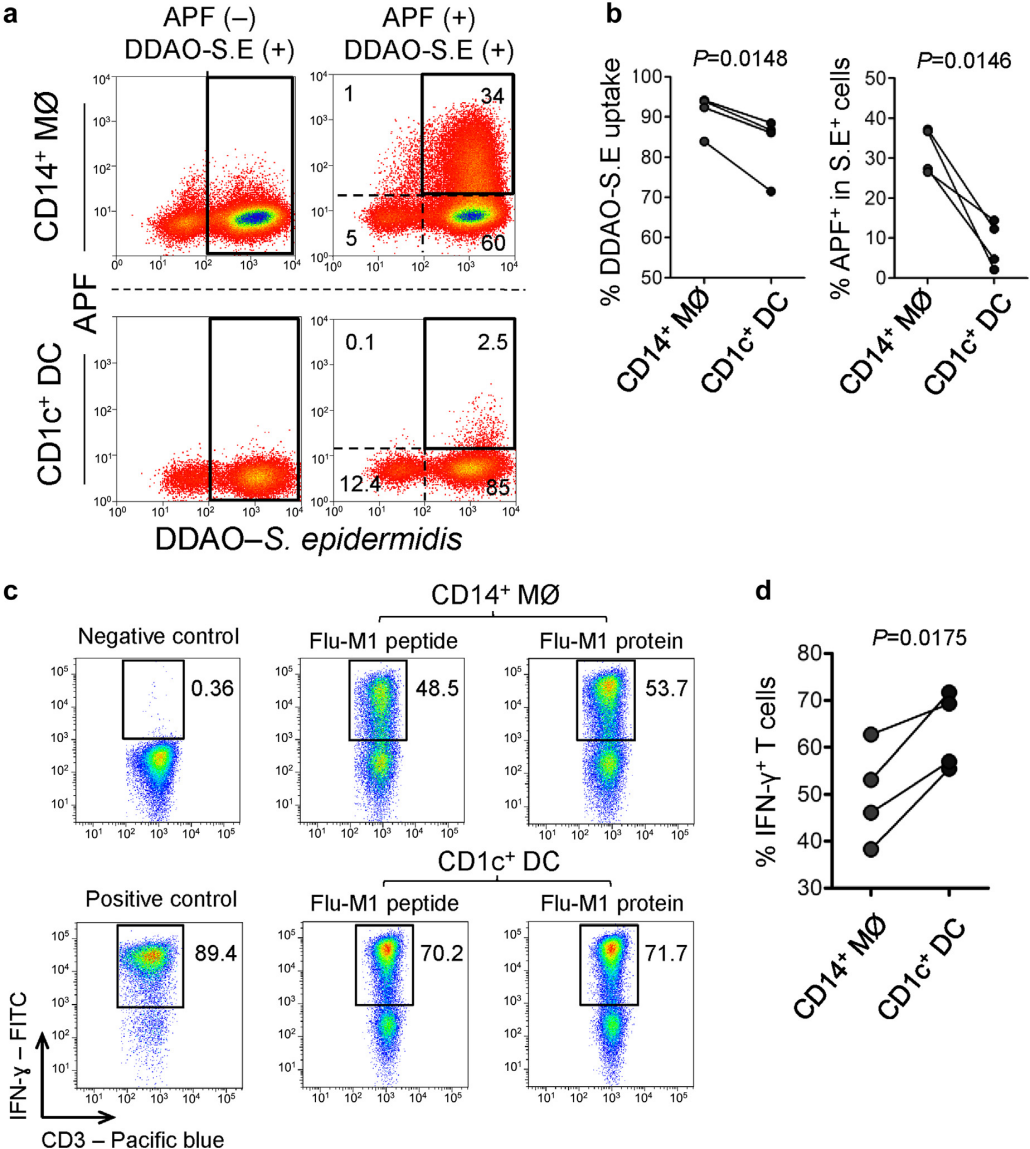
**Functional characterization of peritoneal macrophages and dendritic cells.** (**a**) Representative flow-cytometric density plots showing the generation of reactive oxygen species from peritoneal MØ/DC, detected by 3’-(p-aminophenyl) fluorescein (APF), following challenges with 7-hydroxy-9H-(1,3-dichloro-9,9-dimethylacridin-2-one) (DDAO)-conjugated Staphylococcus epidermidis (S.E) *ex vivo* or cultured in medium only. The percentages of cells found in each of specified gate are indicated. (**b**) Quantification of bacterial uptake by respective monocytic cells (left graph) and positive APF staining (%) within the cells phagocytosing DDAO-S.E (right graph). Data are representative of 4 stable PD patients and were analyzed by paired *t* test. (**c**) Representative density plots show flow-cytometric determination of intracellular interferon (IFN)-γ production by responder CD8^+^ T cells specific for M1p58-66. Plots display responder T cells after co-culture with CD14^+^ MØ or CD1c^+^ DC (from HLA-A2^+^ stable PD patients), which were loaded with soluble Flu-M1 protein, or pulsed with Flu-M1p58–66 peptide (at an MØ/DC:responder ratio of 1:1–2). Phorbol myristate acetate (10 μg/ml) + Ionomycin (1 μM) was used as a positive control. Data are representative of 1 from 4 stable PD patients. (**d**) Quantification of intracellular IFN-γ expression from responder T cells derived from antigen processing and presentation assay based on loading Flu-M1 protein antigen on the respective MØ or DC. Data are representative of 4 stable PD patients and were analyzed by paired *t* test.

To functionally validate our findings, we performed *ex vivo* analysis of peritoneal MØ and DC from infection-free PD effluents. On 7-hydroxy-9H-(1,3-dichloro-9,9-dimethylacridin-2-one)–labeled *S epidermidis* challenge, CD14^+^ MØs displayed higher capacity of phagocytosis as well as generated more reactive oxygen species (detected by 3’-[p-aminophenyl] fluorescein), compared with CD1c^+^ DCs (Figure 4a and 4b). To test their ability to uptake, process, and present specific antigen to responder T cells, a modified *in vitro* assay of cross presentation was conducted.^34^ The results suggested that both cell types are effective antigen-processing and -presenting cells, albeit CD1c^+^ DCs are more efficient than CD14^+^ MØs in this regard (Figure 4c and 4d). To investigate the cellular responses to microbial stimuli, the production of the proinflammatory cytokines (tumor necrosis factor-α, IL-1β, IL-6, IL-12p40/p70) and anti-inflammatory cytokine (IL-10) were measured by intracellular flow cytometry after *ex vivo* stimulation with lipopolysaccharide or *S epidermidis* supernatant^4^ (Supplementary Figure S3A). Both CD14^+^ MØ and CD1c^+^ DC could generate significant amounts of tumor necrosis factor-α, IL-1β, and IL-6, as well as small amounts of IL-10 and IL-12p40/p70 after stimulation (Supplementary Figure S3B), albeit that CD14^+^ MØs generated higher tumor necrosis factor-α and IL-6 levels than CD1c^+^ DCs on either *ex vivo S epidermidis* supernatant or lipopolysaccharide stimulation.

### Heterogeneous macrophage activation and monocyte-to-macrophage maturation in PD patients

**Figure 5:**
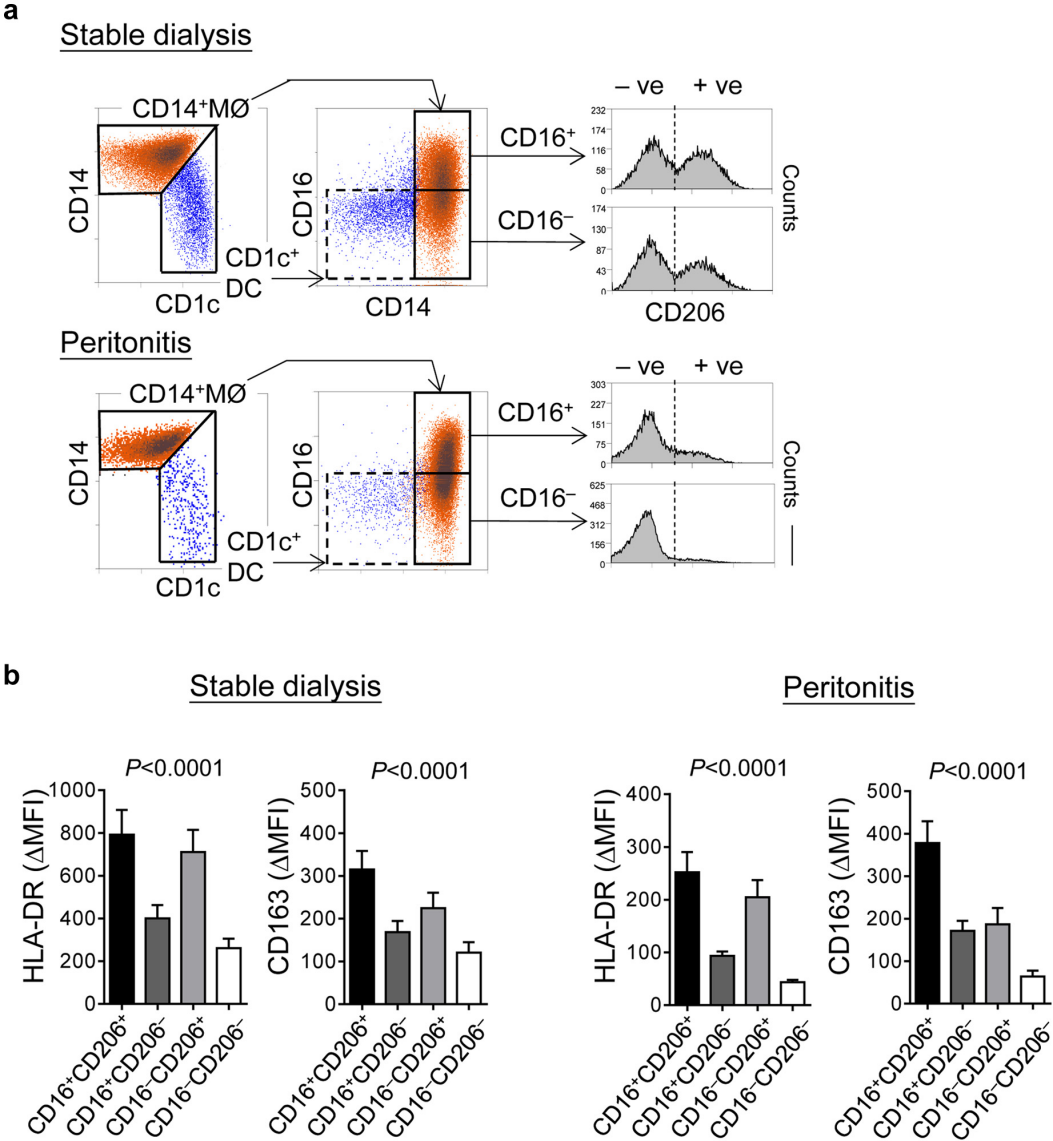
**Heterogeneous activation and maturation of peritoneal macrophages under different clinical settings.** (**a**) Representative flow-cytometric density plots (left) and histogram plots (right) showing the segregation of CD14^+^ MØs (orange) and CD1c^+^ DCs (blue) under stable status (upper panel) and during peritonitis (lower panel). Contrast to CD1c^+^ DCs with CD16^low/‒^ and CD206^high/+^, CD14^+^ MØs could be subgated into CD16^+^ and CD16^‒^, each further stratified into C206^‒^ and CD206^+^, resulting into 4 MØ subtypes. Data are representative of the analysis of 42 stable and 42 peritonitis patients, and the same approach was applied through all analyzed PD samples in this study. (**b**) Bar graphs comparing HLA-DR and CD163 expression, measured as the difference in medium fluorescence intensity (MFI) between receptor-specific and isotype control staining, among individual 4 peritoneal MØ subtypes from PD patients under stable dialysis (left, *n* = 20) and during acute bacterial peritonitis (right, *n* = 20). Data are shown as mean ± SEM and was analyzed by 1-way analysis of variance tests.

Macrophages retain a substantial phenotypic plasticity, reflected by their heterogeneous activation and maturation status under different microenvironments. We observed that CD14^+^ MØs from PD effluent, unlike CD1c^+^ DCs, displayed variable CD16 and CD206 expression (Figure 5a). Both CD16 and CD206 have been adopted as surface markers indicative of macrophage activation or monocyte-to-macrophage maturation.^35^, ^36^, ^37^ Based on these 2 markers, CD14^+^ MØs could be stratified into 4 subtypes: CD16^+^CD206^+^, CD16^+^CD206^‒^, CD16^‒^CD206^+^, CD16^‒^CD206^‒^ (Figure 5a), most likely representing different stages of MØ activation and maturation within the peritoneal cavity. Of note, CD16^+^CD206^+^ subtype expresses the highest levels of HLA-DR and CD163 (“mature phenotype”), whereas CD16^–^CD206^–^subtype expresses the lowest levels of these surface antigens (“immature phenotype”) (Figure 5b). CD16^+^CD206^–^ and CD16^–^CD206^+^ MØs potentially represent cells of intermediate phenotype.

### Distinct patterns of myeloid cell distribution associate with patient outcomes

**Figure 6:**
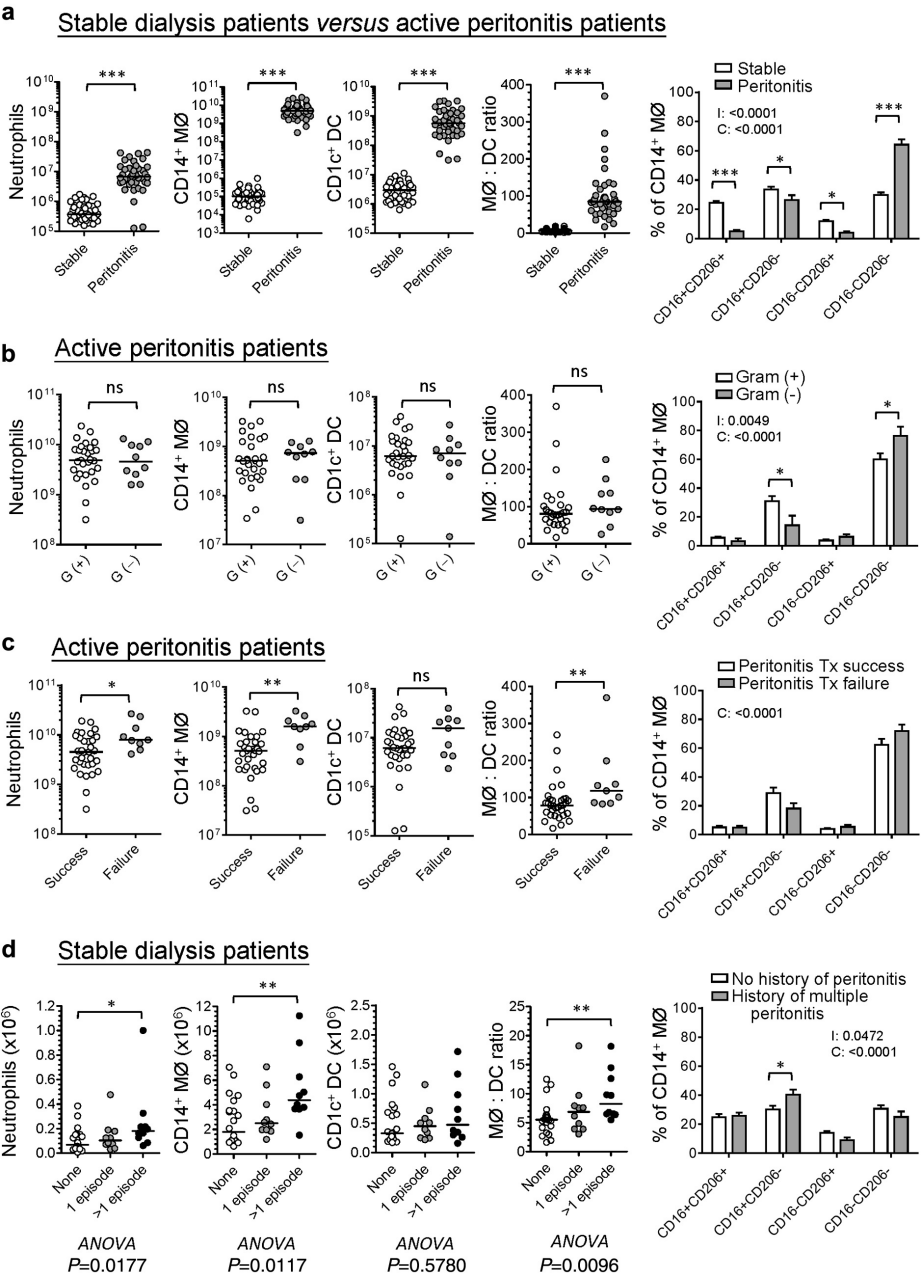
**Distribution of peritoneal myeloid subsets under different clinical settings.** Graphs showing the comparisons of peritoneal neutrophils, CD14^+^ MØs, CD1c^+^ DCs (in actual number), the ratio of MØ to DC, and the percentage of each MØ subtype within CD14^+^ MØs, (**a**) between stable PD patients (*n* = 42) and day 1 bacterial peritonitis patients (*n* = 42). (**b**) Among gram-positive (G[+], *n* = 29), gram-negative (G[‒], *n* = 10), and mixed G(+)/G(‒) peritonitis (*n* = 3). (**c**) Between the peritonitis episodes with treatment success (*n* = 33) and those with treatment failure (*n* = 9). (**d**) Among stable PD patients without history of peritonitis (*n* = 22), those with only 1 episode of peritonitis (*n* = 10) and those with more than 1 episode of peritonitis (*n* = 10). Horizontal bars denote the medium of each group. Data were analyzed for statistical significance by Mann-Whitney *U* tests (2-group comparison) or Kruskal-Wallis tests (3-group comparison) with Dunn’s multiple comparison post-tests (asterisks) indicated where significant. For bar graphs, data were shown as mean, and error bars denote SEM. Data were analyzed by 2-way analysis of variance (ANOVA) tests with Sidak’s multiple comparisons test. I, interaction; C, cell subtype; P, stable patients with or without history of peritonitis; Tx, treatment.

The composition of peritoneal leukocyte subpopulations is altered under different clinical settings.^27^ Here, our data revealed that peritoneal neutrophils, MØs, and DCs significantly increased in the infected fluids from acute bacterial peritonitis, compared with infection-free fluid samples from stable dialysis patients (Figure 6a). With regard to MØ heterogeneity, there was a significantly higher CD16^‒^CD206^‒^ subtype (%) and lower CD16^+^CD206^+^ subtype (%) within the infectious fluids, compared with infection-free fluids (Figure 6a, right panel). Interestingly, in patients with acute bacterial peritonitis, the increase in CD14^+^ MØs was much greater than CD1c^+^ DCs, and the ratio of MØ to DC skewed up significantly in peritonitis samples. There was no difference between gram-positive peritonitis and gram-negative peritonitis in this regard (Figure 6b); however, PD fluids from gram-negative peritonitis contained slightly more CD16^‒^CD206^‒^ MØ and less CD16^+^CD206^‒^ MØ than those from gram-positive peritonitis (Figure 6b, right panel). Importantly, unresolved/refractory peritonitis episodes were associated with significantly higher numbers of peritoneal neutrophils, MØs as well as higher ratio of MØ to DC than successfully treated ones (Figure 6c). There was also no significant difference in MØ heterogeneity between successfully treated peritonitis and failed cases (Figure 6c, right panel). To examine the impact of peritonitis history on the distribution of peritoneal myeloid subsets, we compared stable dialysis patients without history of peritonitis and those with only 1 episode or multiple episodes, showing that there was a higher number of peritoneal MØs and a higher MØ-to-DC ratio in patients with multiple episodes of peritonitis, compared with those without any history of peritoneal infection (Figure 6d). The former also has a significantly higher proportion of CD16^+^CD206^‒^ MØ than the latter (Figure 6d, right panel).

### Myeloid cell distribution and macrophage heterogeneity during longitudinal follow-up in “new-starter” PD patients: impact of catheter surgery and dialysis intervention

**Figure 7:**
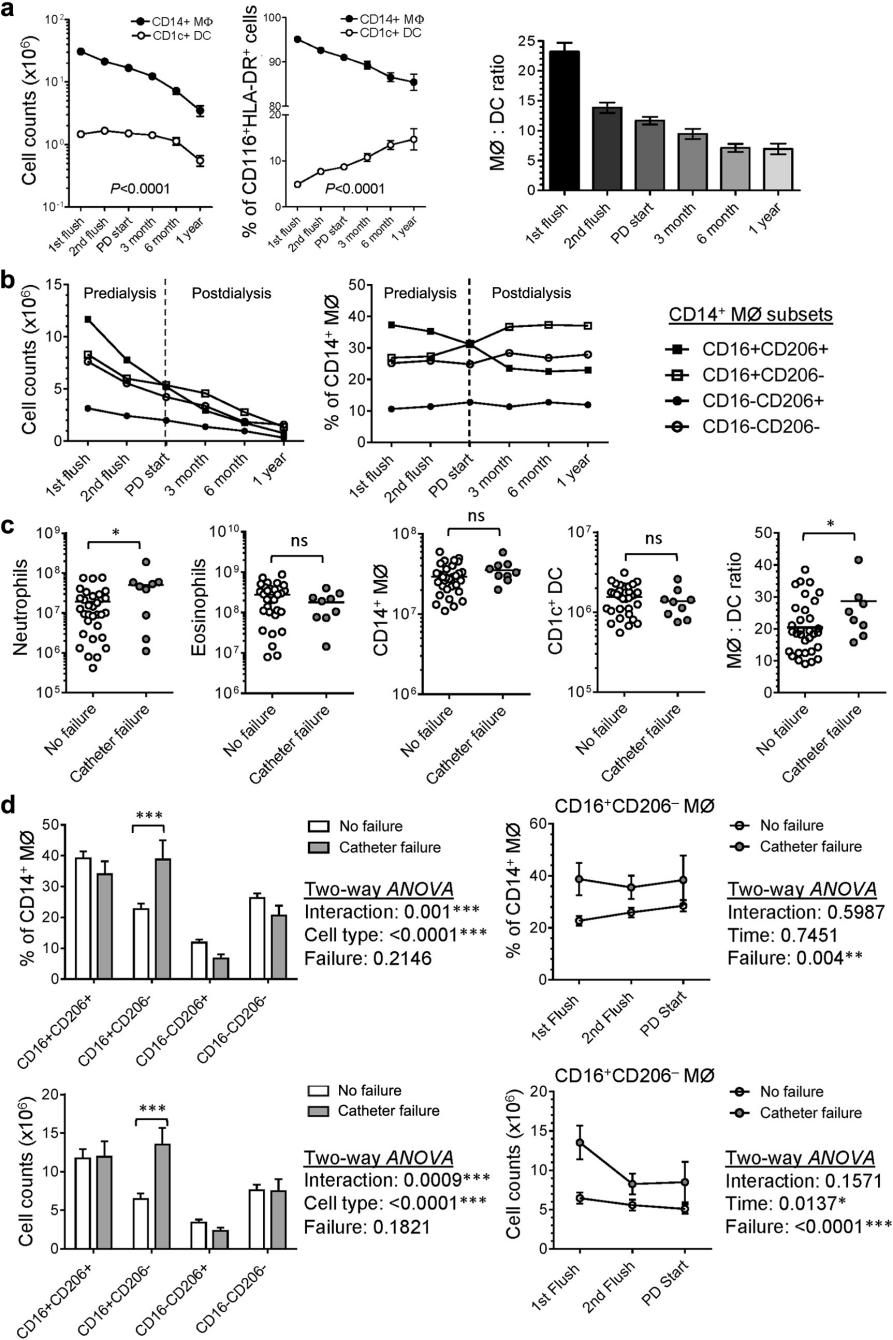
**Myeloid cell distribution and macrophage heterogeneity during longitudinal follow-up in “new-starter” PD patients.** The compositions of mononuclear phagocyte subsets and MØ subtypes in peritoneal effluents have been analyzed from 1 week after PD catheter surgery to the end of the first year of PD treatment. Each patient would have 6 time points of PD fluid sampling if he or she could maintain PD therapy for at least 1 year. In total, 171 samples have been analyzed: 50 samples from *1st flush (1 week after catheter surgery)*, 37 samples from *2nd flush (2–3 weeks after 1st flush)*, 39 samples from *PD start (normally 1 week after 2nd flush)*, 20 samples from *PD for 3 months*, 15 samples from *PD for 6 months*, and 10 samples from *PD for 1 year*. (**a**) Graphs showing the numbers (left) and the proportion (central) of CD14^+^ MØs and CD1c^+^ DCs within peritoneal mononuclear phagocytes as well as the ratio of MØ over DC (right) changed during the first year of PD therapy. (**b**) Graphs showing the cell number (left) and the proportion (right) of each MØ subtype changed during the first year of PD therapy. (**c**) Graphs showing the comparisons of peritoneal neutrophils, MØ and DC (in number) from the first PD flush effluents between patients without (*n* = 32) and with catheter failure (*n* = 9). Horizontal bars represent the mean. Data were analyzed by Student *t* test (if parametric) or Mann-Whitney *U* test (if nonparametric). (**d**) (Left panels) Graphs showing the proportion (upper) and the actual number (lower) of each MØ subtype from the *1st PD flush* of “new-starter” patients without (*n* = 32) and with catheter failure (*n* = 9). Data were shown as mean ± SEM. Data were analyzed by 2-way ANOVA tests with Sidak multiple comparisons test. (Right panels) Graphs depicting the change of the proportion (upper) and the actual number (lower) of CD16^+^CD206^‒^ MØ subtype from the *1st PD flush* to *PD start* of “new-starter” patients without (*n* = 32 for *1st flush*, *n* = 25 for *2nd flush*, *n* = 27 for *PD start*) and with catheter failure (*n* = 9 for *1st flush*, *n* = 4 for *2nd flush*, *n* = 4 for *PD start*). Data are shown as mean ± SEM. Data were analyzed by 2-way analysis of variance (ANOVA) tests.

To understand how the patient’s peritoneal immune system responds to an acute insult (catheter implantation surgery) and the subsequent intervention by continuous dialysis exchange, we longitudinally followed a cohort of 50 “new-starter” PD patients for at least 1 year (Supplementary Figure S4). Based on a similar flow-cytometry approach, we were able to phenotypically identify distinct myeloid subsets including neutrophils, eosinophils, and mononuclear phagocytes (MØ/DC) within the serial collections of PD fluids (Supplementary Figure S5). Despite general decline in the numbers of all myeloid subsets along the course of PD therapy, the proportion of peritoneal mononuclear phagocytes increased, especially after continuous dialysis started (Supplementary Figure S6A and S6B). Intriguingly, CD14^+^ MØ decreased continuously along the course of PD therapy, whereas CD1c^+^ DC maintained a stable number during the predialysis as well as early dialysis period, then declined at a relatively slow rate as dialysis continued, hence resulting in the relative increase in the percentage of CD1c^+^ DC recoverable from the tissue (Figure 7a). The ratio of CD14^+^ MØ to CD1c^+^ DC decreased during the predialysis period and gradually reached stable status after 6-month dialysis (Figure 7a, right panel). Within the CD14^+^ MØ, the main alteration along the course of PD therapy was the increase in the proportion of CD16^+^CD206^‒^ MØ but decrease in CD16^+^CD206^+^ mature subtype (Figure 7b). Irreversible catheter dysfunction/obstruction is a major cause for early technique failure (< 3-month dialysis), as shown in Supplementary Figure S4. Interestingly, compared with patients without catheter failure, those with catheter failure had significantly higher numbers of peritoneal neutrophils as well as higher ratio of MØ to DC (Figure 7c) from their first PD flush. Additionally, there was a significantly higher proportion and number of CD16^+^CD206^‒^ MØ subtype in patients with catheter failure at first PD flush, and also during the predialysis period (Figure 7d).

## Discussion

This study has demonstrated that human peritoneal mononuclear phagocytes from PD patients are a heterogeneous population. Distinct peritoneal monocytic subpopulations most likely represent different developmental origins, differentiation pathways, and activation/maturation states. Importantly, for the first time, we could identify human peritoneal DCs within the PD effluent, which are morphologically, phenotypically, transcriptomically and functionally distinct from the MØs. Our observations also suggested that the alterations of peritoneal MØ/DC distribution and MØ activation/maturation status may indicate a unique scenario and/or severity of i.p. inflammation, which in turn can be linked to patients’ clinical outcomes.

**Figure 8:**
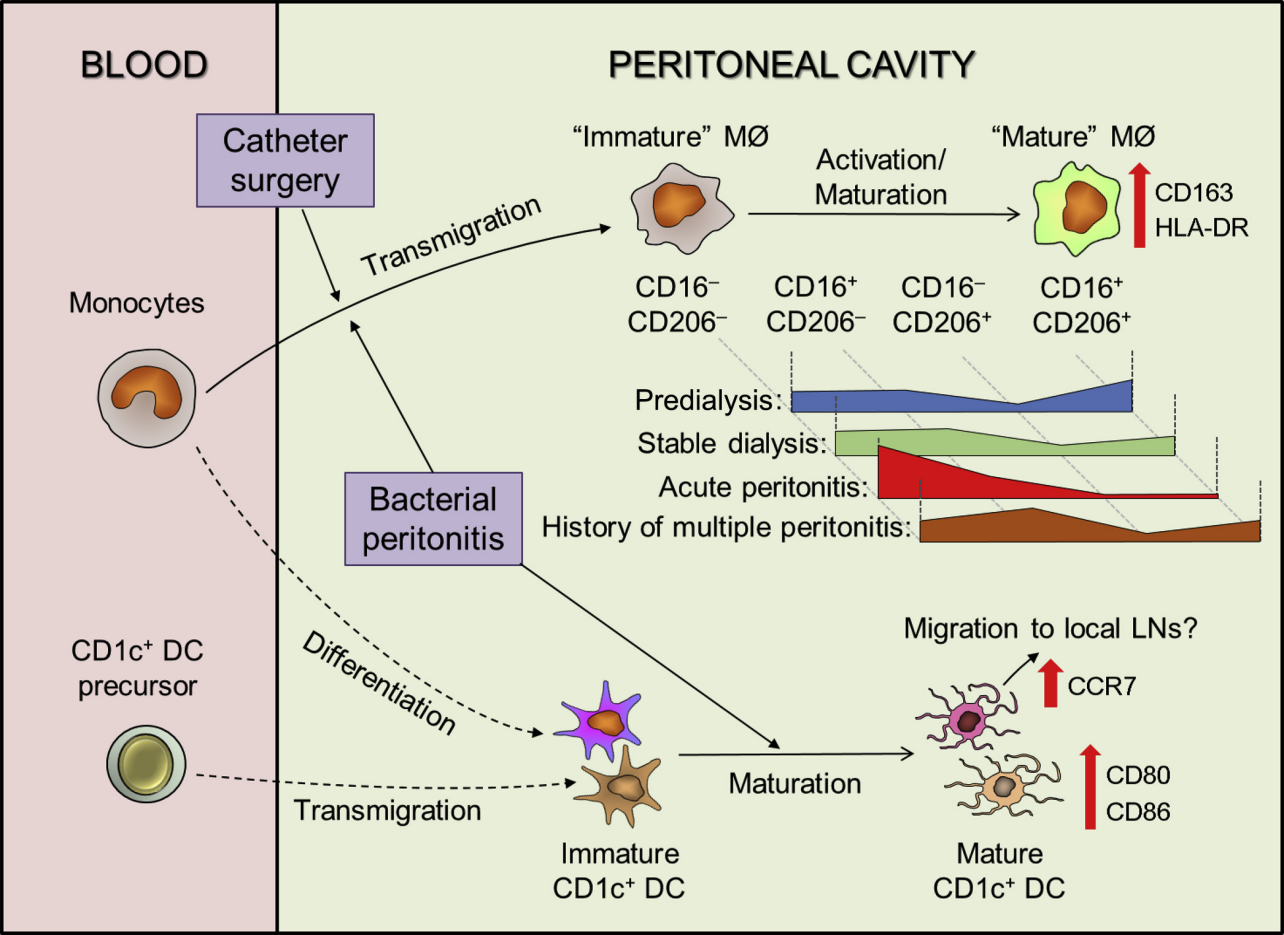
**Summary of cellular kinetics of peritoneal MØ and DC under stable dialysis and on bacterial peritonitis.** Schematic diagram depicting the proposed cellular kinetics of peritoneal MØs and DCs under stable dialysis and on bacterial peritonitis. Peritoneal mononuclear phagocytes from PD effluent comprise CD14^+^ MØs (predominant population) and CD1c^+^ DCs. Under stable status, PD fluid exchange would diminish the resident population within the peritoneal cavity. Blood monocytes were then recruited into the peritoneal cavity, where they undergo maturation process (represented by increased expression of HLA-DR, CD163, and CD206). However, this process is hindered by the dialysis exchange (washing out the mature MØs leading to a relative enrichment of the cavity with the newly recruited immature ones), perhaps compounded by dilution of cytokine/growth factor essential for MØ maturation. Acute peritonitis is associated with a marked elevated influx of immature MØ in the context of continued dialysis. On the other hand, peritoneal CD1c^+^ DCs could be supplied by 2 sources: recruitment of blood CD1c^+^ DC precursors and *in situ* monocyte/MØ-to-DC differentiation. On bacterial peritonitis, the relatively immature peritoneal DCs become activated and acquire a more mature phenotype (represented by increased expression of CD80 and CD86). Although some mature DCs may stimulate peritoneal naïve T cells locally, others bearing with upregulated CCR7 expression may migrate to local lymph nodes to initiate a systemic adaptive immune response. Meanwhile, monocytes are rapidly recruited into the peritoneal cavity, where (alongside neutrophils) their functions likely include execution of the bactericidal activity and clearance of apoptotic cells and tissue debris.

Previous studies in PD patients mainly focused on peritoneal MØ biology (mainly CD14^+^ cells) and only a very few studies attempted to address peritoneal DCs, with no clear identification of this important cell type. Our application of multicolor flow cytometry has identified a CD1c^+^ DC population within the PD effluent. These, not only display distinctive surface marker profiles to CD14^+^ MØ, but have unique transcriptional profiles, illustrating their specialized functional properties. These data have provided new insights into their individual roles in the regulation of peritoneal immunity against invading pathogens. Briefly, blood monocytes are rapidly recruited into peritoneal cavity on peritonitis (Figure 8), presumably depending on the chemotactic gradient generated by the inflamed peritoneum, including, for example, C-C motif chemokine ligand 2/Monocyte chemoattractant protein-1.^38^ These newly arrived monocyte/MØs play a key effector role in bacterial eradication; produce inflammatory mediators, such as tumor necrosis factor-α, IL-1β, IL-6; and possibly relate to severe tissue inflammation and adverse outcomes (see later in this article). On arrival, these monocyte-derived MØs undergo a maturation process (increased expression of CD206, CD163, and HLA-DR), which is potentially hampered by the commencement and continuation of dialysis exchange, as shown by data from the “new-starter” patient cohort. With regard to peritoneal CD1c^+^ DCs, 2 potential origins are identified: direct migration of blood CD1c^+^ DC precursors, and monocyte-to-DC differentiation (monocyte-derived DCs) (Figure 8). Without the ability to perform fate-mapping studies in patients, we rely on the nomenclature classifications of Guilliams *et al.*^39^ to define our CD1c^+^ DC population. The expression of CD1c, CD1a, CD11b, CD206 (confirmed by flow-cytometry and microarray analysis) and high expression of C-X3-C motif chemokine receptor 1 (microarray data, not shown), argue against a conventional/classical DC1 assignment. Additionally, the very low-low CD14, CD16, CD64, MERTK (the latter from comparison of microarray data to CD14^+^ MØ [Supplementary Figure S2]), and relatively high Tool-like receptor 3 expression (Supplementary Figure S2) support a cDC2 assignment instead of monocytic origin. This DC versus monocytic assignment is also supported by the relative expression of FLT3, IRF4, and MAFB (Figure 2b). Interestingly, our recent identification of an analogous population of antigen-presenting cells in mice permitted fate-mapping and factor-dependency studies that also supported the assignment of the cDC2 nomenclature.^17^

Intriguingly, the increase in CD1c^+^ DC number is very limited during peritonitis, compared with CD14^+^ MØs. One possible explanation is that the local microenvironment may favor monocyte influx, rather than DC precursor migration and/or *in situ* monocyte-to-DC differentiation. Hence, the ratio of MØ to DC drastically increases on peritonitis. Additionally, CD1c^+^ DCs maintained a relatively stable number in the predialysis phase and appeared more resistant to the dialysis depletion, which was in contrast to CD14^+^ MØs. These distinctive kinetics of CD14^+^ MØs and CD1c^+^ DCs during the course of PD therapy, as well as peritonitis episodes, has implied a different recruitment or differentiation processes, driven by local cytokines/chemokines under steady state and during inflammation. Nonetheless, these DCs may acquire a mature phenotype on infectious challenge, resulting in increased antigen-presenting capability (upregulated CD80/CD86 expression) for T-cell stimulation locally, or systemically through migration of CCR7^+^ DCs to draining lymph nodes. Notably, a previous study within our unit revealed that there is a substantial enrichment of the effector memory T-cell subset within PD effluent from stable PD patients^40^; perhaps this could be linked to the role of peritoneal DCs in establishing local adaptive immunity. Related, a recent study investigating peritoneal MØ/DC subsets within tumor ascites from patients with advanced ovarian cancer also identified CD14^+^ MØs and CD1c^+^ DCs, through a different flow-cytometric gating approach.^41^ Comparison of the transcriptome datasets demonstrates important correlations (Supplementary Figure S7).

The present study indicates that changes in peritoneal MØ/DC distribution and MØ activation/maturation status in PD patients may indicate the severity of i.p. inflammation. For example, patients with peritonitis failure had higher ratios of MØ to DC than those with good prognosis. Similarly, “new-starter” PD patients with catheter failure had higher ratios of MØ to DC than those without failure. These findings suggested that higher ratios are associated with more severe i.p. inflammation. The trend also could be observed in stable patients with a history of multiple peritonitis episodes, when compared with patients with no history of peritonitis. Notably, a recent observational study revealed that stable dialysis patients with a history of peritonitis had higher levels of dialysate MCP-1, compared with those without any history of peritonitis.^42^ Hence, the increased MØ accumulation (and also the MØ-to-DC ratio) within PD effluent may reflect chronic peritoneal inflammation after recurrent peritonitis. These recruited MØs could generate significant amounts of proinflammatory cytokines on infectious challenges, and so potentiate the development of membrane fibrosis, similar to the results found in our Th1-driven repeat-hit *S epidermidis* supernatant-induced experimental peritoneal fibrosis model.^6^ Furthermore, heterogeneous MØ activation/maturation status was observed under different clinical scenarios and potentially related to patient outcomes. For example, there is a trend of increased proportion of CD16^‒^CD206^‒^ MØ subtype in gram-negative peritonitis and failed peritonitis treatment, whereas increased proportion of CD16^+^CD206^‒^ MØ subtype in “new-starter” patients with catheter failure and stable patients with history of repeated peritonitis. Interestingly, after catheter implantation, but before continuous dialysis begins, the CD16^+^CD206^+^ is seen to be the largest MØ subset, reflecting an accumulation of mature cells. In the future, it would be interesting to investigate how these altered MØ/DC distributions and MØ activation/maturation states correlate to membrane dysfunction/fibrosis in patients receiving long-term PD. Indeed, the importance of this connection has been addressed very recently.^43^ By transcriptional profiling of peritoneal tissues from PD patients with encapsulating peritoneal sclerosis, those without encapsulating peritoneal sclerosis or uremic patients without history of PD, the pathway analysis of the differentially expressed genes has revealed enrichment in several pathways relating to MØ/DC function and activation/maturation in patients developing encapsulating peritoneal sclerosis.

In summary, the present study has provided a broad insight into peritoneal MØ/DC biology in PD patients. Through deciphering the complexity of phenotypic and functional heterogeneity of these cells, the specific role of individual MØ/DC subsets in the context of peritonitis as well as alterations after catheter implantation and dialysis intervention have been demonstrated, and links between MØ/DC biology and patient outcome described.

## Methods

### Patients

Patients were recruited from the PD unit at University Hospital of Wales in Cardiff, UK. A total of 42 episodes of acute bacterial peritonitis were included in this study. The causative organism(s) and the treatment outcome of peritonitis episodes were recorded. The outcome of peritonitis treatment was categorized into “treatment success” or “treatment failure” (defined as peritonitis-related mortality or technique failure with permanent transfer to hemodialysis). Meanwhile, 42 stable patients under maintenance PD therapy for more than 6 months were enrolled for comparisons. These patients were free from peritonitis for at least 3 months at the time of PD fluid sampling. Additionally, a cohort of “new-starter” PD patients (*n* = 50) was longitudinally followed for at least 1 year. Patient outcomes such as nondeath technique failure, deaths, or receiving transplantation were documented. This study was undertaken according to principles described in the Declaration of Helsinki and under the local ethical guidelines (Bro Taf Health Authority, Wales) and approved by the South East Wales Local Ethics Committee (COREC:04WSE04/27). All patients provided written informed consent.

### Isolation of peritoneal cells

Peritoneal cells were harvested either from “cloudy bags” of day 1 peritonitis, or from uninfected overnight dwell bags as previously described.^27^ Cells were counted by hemocytometer and the leukocyte composition was assessed by flow cytometry (see the following sections).

### Flow cytometry and cell sorting

Cells were stained with LIVE/DEAD Fixable Aqua Stain kit (Life technologies, Carlsbad, California, USA), before blocking (1% vol/vol normal mouse serum) and staining of anti-human monoclonal antibodies (together with isotype controls) (Supplementary Table S1). Cells were acquired on either the Cyan ADP (Beckman-Coulter, Brea, California, USA) or FACSCanto II (BD Biosciences, San Diego, California, USA) flow cytometers with Summit software (Beckman-Coulter) or FlowJo (TreeStar, Ashland, Oregon, USA). The respective MØs and DCs were purified by flow cytometry (FACSAria III; BD Biosciences) based on the same gating strategy for MØ/DC subset identification. The purified subsets were immediately used in morphological images, functional assays, and microarray analyses (see later in this article).

### Morphological analysis

Cells were cytospun (Cytospin 3, 300 rpm for 5 minutes; Shandon, Runcorn, UK), air-dried, stained with Microscopy Hemacolor (Merck, Millipore, Billerica, Massachusetts, USA), visualized on a Leica DMLB microscope with DFC490 camera (Leica, Wetzlar, Germany) and processed using QWin Software (Leica).

### RNA isolation and microarray analysis

Total RNA was extracted from purified MØs or DCs using miRNeasy micro kit (Qiagen, Hilden, Germany), according to manufacturer’s protocol. After reverse-transcription reactions, double-strand DNA was hybridized on the Affymetrix (Santa Clara, California, USA) GeneChip Human Gene 1.0 ST Array. Detailed information regarding microarray analysis is described in the Supplementary Material.

### *Ex vivo* phagocytosis assay and respiratory burst activity

Cells were pre-incubated with 3’-(p-aminophenyl) fluorescein (5 μM final concentration; from Life technologies) for 30 minutes at 37 ^o^C, 5% CO_2_ in the dark, before the addition of 7-hydroxy-9H-(1,3-dichloro-9,9-dimethylacridin-2-one)–conjugated *S epidermidis* (∼10^8^ colony-forming units) for 30 minutes at 37 ^o^C, 5% CO_2_ in the dark. After wash, cells were stained and analyzed with flow cytometer as described previously.

### Antigen-processing and -presenting assay

Assays were performed as previously described with slight modifications^34^ (detailed in Supplementary Material). Briefly, MØs or DCs were loaded with either recombinant Influenza M1 protein (1 μM) or M1p58–66 peptide (0.1 μM), before co-culturing with responder CD8+ T cells. Responder T cells alone and with phorbol myristate acetate (10 μg/ml) + ionomycin (1μM) were used as negative and positive controls, respectively. The co-cultures were incubated for 5 hours at 37°C in the presence of Brefeldin-A (10 μg/ml; Sigma-Aldrich, St. Louis, Missouri, USA). Cells were then stained with surface antibodies, fixed and permeabilized for intracellular staining with anti–interferon-ɣ-FITC and analyzed with flow cytometry as described previously.

### Statistical analysis

Statistical analyses were conducted by using the GraphPad Prism (GraphPad Software, San Diego, California, USA). The statistical tests used are indicated as appropriate within the text. *P* values are denoted or summarized as follows: *P ≤ 0.05; **P ≤ 0.01; and ***P ≤ 0.001. All analyses performed were 2-tailed.

## Disclosure

All the authors declared no competing interests.

## Supplementary Material

Supplementary Methods

Figure S1 Peritoneal CD14^+^ cells and CD1c^+^ cells involved in distinct canonical pathways for the up- and downregulated genes. Ingenuity pathway analysis of the differentially expressed genes comparing peritoneal CD14^+^ cells and CD1c^+^ cells purified from 5 stable PD patients. Differentially expressed genes were identified using right-tailed Fisher exact test sorted for *P* < 0.05 and fold change ≥2 between the 2 populations. Based on these criteria, 192 upregulated and 236 downregulated genes (CD14^+^ cells vs. CD1c^+^ cells) were identified. Graphs show the enriched pathways for the up- (**A**) and downregulated (**B**) genes. Blue bars denote the *P* values (−log10) for each pathway enrichment; –log (*P* value) cutoff of 1.3 was applied, meaning that pathways with a *P* value ≥ 0.05 are hidden. The orange squares indicate the ratio of the number of up- and downregulated genes to the total number of molecules within that pathway represented by the connecting orange line. The horizontal solid orange line corresponds to the *P* = 0.05 threshold.

Figure S2 Peritoneal MØ and DC display distinct transcriptomic signatures. Heatmaps representing the relative expression of selective genes encoding key molecules involving major immune functions such as Toll-like receptors (TLR), c-type lectin receptors (CLR), scavenger receptors, Fc receptors, chemokine/receptors, cytokine/receptors, and molecules responsible for antigen presentation. Differentially expressed genes selected for analysis were based on fold change ≥2, either CD14^+^ cells versus CD1c^+^ cells or vice versa, then clustered on heat maps.

Figure S3 Cytokine production in response to *ex vivo* microbial product stimulation. (**A**) Representative density plots illustrating the flow-cytometric determination of intracellular cytokine production by peritoneal MØ/DC from stable PD patients, after *ex vivo* stimulation with *S epidermidis* supernatant (SES) (1:10 dilution) or lipopolysaccharide (LPS) for 4 hours (tumor necrosis factor [TNF]-α, IL-1β, IL-6) or 24 hours (IL-10), or 22 hours (IL-12p40/p70, in addition to 2 hours of interferon [IFN]-γ priming), in parallel with medium only as unstimulated control. Data are derived from 1 patient representative of 7 stable patients giving similar results. (**B**) Bar graphs showing the quantitative comparisons of the intracellular cytokine production between peritoneal CD14^+^ MØs and CD1c^+^ DCs. Cells were cultured in medium alone or stimulated *ex vivo* with SES or LPS as described previously. Positive intracellular cytokine production was determined by comparison with isotype controls. Data represent mean ± SEM and are derived from 7 stable PD patients. Statistical analysis was performed by paired *t* tests.

Figure S4 Flow diagram of longitudinal outcomes of new-starter PD patients during 1- year follow-up. A total of 50 new-starter PD patients was enrolled in this study. The reasons for dropout from this cohort during 1-year follow-up period included receiving kidney transplantation (*n* = 12), transferring to other institutions (*n* = 1), nondeath technique failure (*n* = 19), death (*n* = 3). The number in each set of parentheses denotes the number of patients under each defined category.

Figure S5 Flow-cytometric gating strategies to identify different peritoneal myeloid subsets from PD effluent fluids. Density plots show myeloid cells were pregated on CD116^+^ populations after exclusion of doublets, cellular debris, and dead cells. Within these cells, monocytic cells could be readily identified as HLA-DR^+^CD14^+/low/–^. Granulocytes were HLA-DR^–^CD14^–^. Other useful surface markers, such as CD16, CD15, CD66, CCR3, siglec-8, are applied for confirmation of neutrophils (CD16^+^CD15^+^CD66^+^) and eosinophils (CD16^–^CCR3^+^siglec-8^+^). Data represent the analysis of peritoneal effluent cells from the first flush bag of a new-starter PD patient. NK, natural killer.

Figure S6 Changes in the number and the proportion of major peritoneal myeloid subsets during the first year of PD therapy. (**A**) Data showing the numbers of total peritoneal cells, neutrophils, eosinophils, and monocytic cells changed during the 1-year follow-up period. Data were representative of 171 PD samples from 50 patients at 6 time points (50 samples from *1st flush*, 37 samples from *2nd flush*, 39 samples from *PD start*, 20 samples from *PD for 3 months*, 15 samples from *PD for 6 months,* and 10 samples from *PD for 1 year*). Horizontal bar in each group represents the mean. (**B**) Graphs showing the numbers (left) and the proportion (right) of peritoneal neutrophils, eosinophils, and monocytic cells changed during the 1-year follow-up period. Data were derived from the same samples from (**A**). Error bars in each group denote the SEM.

Figure S7 Comparison of 2 microarray datasets. The array data were generated (or downloaded) as described in the methods section. Our data were compared with that of Segura *et al.*^41^ (**A**) We compared the expression of the probesets that were significantly regulated in both our study and the Segura *et al.*^41^ dataset (between the 2 peritoneal subsets reported) (using 2 FDR-corrected *P*-value thresholds for regulation in the datasets as indicated). There was a high correlation between the expression patterns reported by Segura *et al.*^41^ and in our dataset, indicating likely overlap in the cellular identities. (**B**) The same data were also represented as a volcano plot to demonstrate fold change and *P* value in our dataset.

Table S1 Antibodies used in this study were purchased from BD Biosciences, Biolegend, eBioscience, or R & D Systems. Biotin-conjugated antibodies were detected with phycoerythrin (PE)-TexasRed-, allophycocyanin-, Peridinin-chlorophyll-protein complex
