# Supplementary material for: Peritoneal macrophage heterogeneity is associated with different peritoneal dialysis outcomes
Source: Kidney Int. 2017 May;91(5):1088–103. doi: 10.1016/j.kint.2016.10.030 (PMC5402633; doi:10.1016/j.kint.2016.10.030)
Supplement: Supplementary Methods [file mmc1.docx]

**Supplemental methods**

**Microarray analysis**

For microarray analysis, raw affymetrix data files (CEL files) were imported into an in-house analysis pipeline written in R (version 3.1.1) using packages published in Bioconductor,^1^ namely limma^2^ and oligo.^3^Data were background corrected, log2 transformed and quantile normalized using RMA (robust multi-array average).^4^ Quality control of raw and normalized data was performed to identify potential array/sample outliers, using PCA (principal component analysis, prcomp) and hierarchical clustering (hclust).  No outliers were observed and data were shown to cluster by cell type at this point all data were used for downstream analysis. Differentially expressed genes and transcripts was performed using a Baysian approach,^2^ and p-values were corrected for multiple testing using Benjamini-Hochberg (false discovery rate). The Affymetrix data of Segura et al.,^5^ were downloaded from GEO (GSE40484) and processed in the same way. Probesets were annotated using MetaCore^TM^ (Thomson Reuters) and canonical pathway analysis was performed using MetaCore^TM^ (Thomson Reuters) and Ingenuity Pathway Analysis^TM^ (IPA). Hierarchical clustering and heat maps were generated by Genesis software.^6^

**Bibliography**

1. Gentleman RC, Carey VJ, Bates DM, Bolstad B, Dettling M, Dudoit S, Ellis B, Gautier L, Ge Y, Gentry J, Hornik K, Hothorn T, Huber W, Iacus S, Irizarry R, Leisch F, Li C, Maechler M, Rossini AJ, Sawitzki G, Smith C, Smyth G, Tierney L, Yang JY, Zhang J: Bioconductor: Open software development for computational biology and bioinformatics R. *Genome Biology* 5:R80, 2004
2. Ritchie ME, Phipson B, Wu D, Hu Y, Law CW, Shi W, Smyth GK: Limma powers differential expression analyses for RNA-sequencing and microarray studies. *Nucleic Acids Research* 43:e47, 201
3. Carvalho BS and Irizarry RA: A framework for oligonucleotide microarray preprocessing. *Bioinformatics*, 26:2363-2367, 2010
4. Segura E, Touzot M, Bohineust A, Cappuccio A, Chiocchia G, Hosmalin A, Dalod M, Soumelis V, Amigorena S: Human inflammatory dendritic cells induce Th17 cell differentiation. *Immunity* 38:336–348, 2013
5. Irizarry RA, Hobbs B, Beazer-Barclay YD, Antonellis KJ, Scherf U, Speed TP: Exploration, normalization and summaries of high density oligonucleotide array probe level data. *Biostatistics* 4:249-264, 2003
6. Sturn A, Quackenbush J, Trajanoski Z: Genesis: cluster analysis of microarray data. *Bioinformatics* 18:207-208, 2002

**Antigen processing and presenting assay**

Stable PD patients were screened by flow cytometry for their HLA-A2 status (anti-HLA-A2-APC, clone: BB7.2, eBiosicences). Antigen cross-presentation assays were performed as previously described with modifications*.*^1^ The purified peritoneal APC subsets were cultured in 96-well round-bottomed plates (5x10^4^-2x10^5^ cells/well). M1-specific CD8^+^ T cells were generated by stimulating PBMCs with 0.1 nM M1p58-66. Resting cells with >95% M1p58-66-specific cells were used for assays, as determined by PE-tetramer staining (homemade). Recombinant Influenza M1 protein (1 μM) was added to the APCs for 20 h at 37 °C, 5% CO_2_. APCs were washed extensively before co-culturing with responder T cells at a ratio of 1:1-2 (APCs: responder T cells). M1p58-66 peptide (0.1 μM) was added to the APCs for 1 h at 37°C prior to co-cultured with responder T cells. Responder cells alone and with 10 µg/ml PMA and 1 µM ionomycin were used as negative and positive controls, respectively. The co-cultures were incubated for 5 h at 37°C, 5% CO_2_ in the presence of Brefeldin-A (10 µg/ml; Sigma-Aldrich). In brief, the cells were then stained with LIVE/DEAD Fixable Aqua Stain kit (Life technologies), blocked for 15 minutes with normal human IgG (0.1 mg/ml, KIOVIG, Baxter) and then stained with surface antibodies for 30 minutes on ice. The cells were fixed and permeabilised for intracellular staining with anti-IFN-ɣ-FITC (B27, BD Biosciences) for 30 minutes on ice in the dark. The samples were acquired on either the FACSCanto II or CyAn ADP flow cytometers and analyzed with FlowJo (TreeStar) or Summit software.

**Bibliography**

1. Meuter S, Eberl M, Moser B: Prolonged antigen survival and cytosolic export in cross-presenting human gammadelta T cells. *Proc Natl Acad Sci U S A* 107:8730–8735, 2010

***Ex Vivo* Stimulation and Cytokine Responses**

Cells were stimulated with SES (10% v/v) or LPS (100 ng/ml final concentration; from *Salmonella enterica* serotype typhimurium, Sigma) or left unstimulated (medium only) in the presence of GolgiStop™ (2 μM final concentration; BD Bioscience) for 4 h (TNF-α, IL-1β, IL-6) or 24 h (IL-10) at 37 °C, 5% CO_2_. For IL-12p40/p70 assay, cells were primed for 2 hours with recombinant human IFN-γ (10 ng/ml final concentration; PeproTech), then activated with IFN-γ (10 ng/ml final concentration) and either SES (10% v/v) or LPS (100 ng/ml final concentration) in the presence of GolgiStop™ (2 μM final concentration; BD Bioscience) for an additional 22 hours at 37 °C, 5% CO_2_. After incubation, cells were carefully recovered, stained with surface antibodies, fixed and permeabilized, and then stained with anti-human cytokine antibodies (**Supplemental Table S1**). Cells were analyzed by flow cytometry as previously described
