# Supplementary material for: Peritoneal macrophage heterogeneity is associated with different peritoneal dialysis outcomes
Source: Kidney Int. 2017 May;91(5):1088–103. doi: 10.1016/j.kint.2016.10.030 (PMC5402633; doi:10.1016/j.kint.2016.10.030)
Supplement: Figure S1 — Peritoneal CD14+ cells and CD1c+ cells involved in distinct canonical pathways for the up- and downregulated genes. Ingenuity pathway analysis of the differentially expressed genes comparing peritoneal CD14+ cells and CD1c+ cells purified from 5 stable PD patients. Differentially expressed genes were identified using right-tailed Fisher exact test sorted for P < 0.05 and fold change ≥2 between the 2 populations. Based on these criteria, 192 upregulated and 236 downregulated genes (CD14+ cells vs. CD1c+ cells) were identified. Graphs show the enriched pathways for the up- (A) and downregulated (B) genes. Blue bars denote the P values (−log10) for each pathway enrichment; –log (P value) cutoff of 1.3 was applied, meaning that pathways with a P value ≥ 0.05 are hidden. The orange squares indicate the ratio of the number of up- and downregulated genes to the total number of molecules within that pathway represented by the connecting orange line. The horizontal solid orange line corresponds to the P = 0.05 threshold. [file mmc2.docx]

**Figure S1.**

*Liao et al., Suppl Figure 1*

Up-regulated pathways in CD14^+^ cells vs CD1c^+^ cells

**A**

**B**

Down-regulated pathways in CD14^+^ cells vs CD1c^+^ cells

**
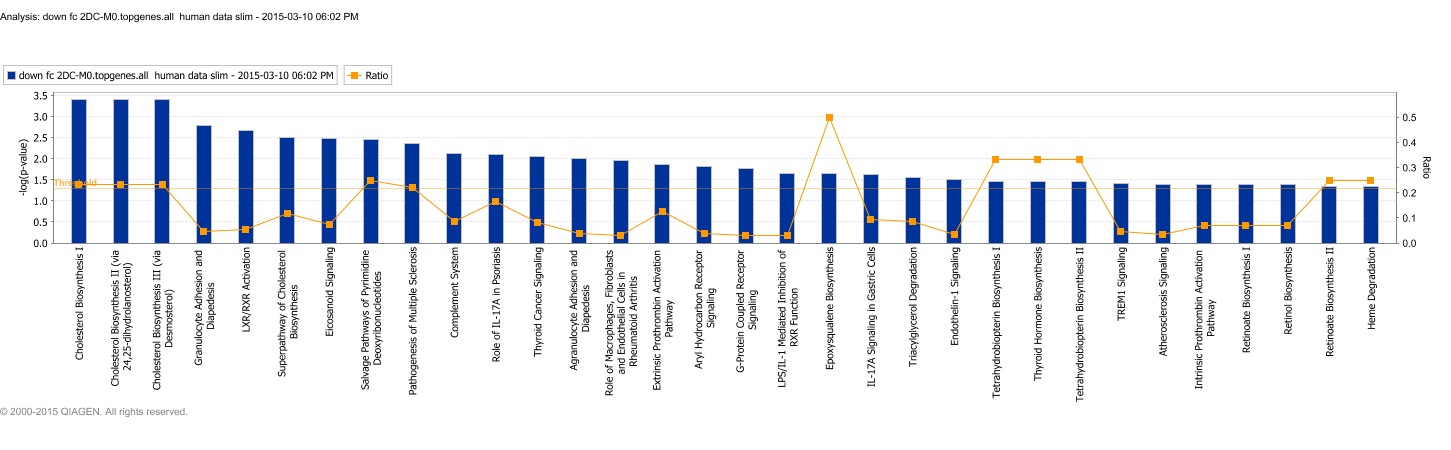

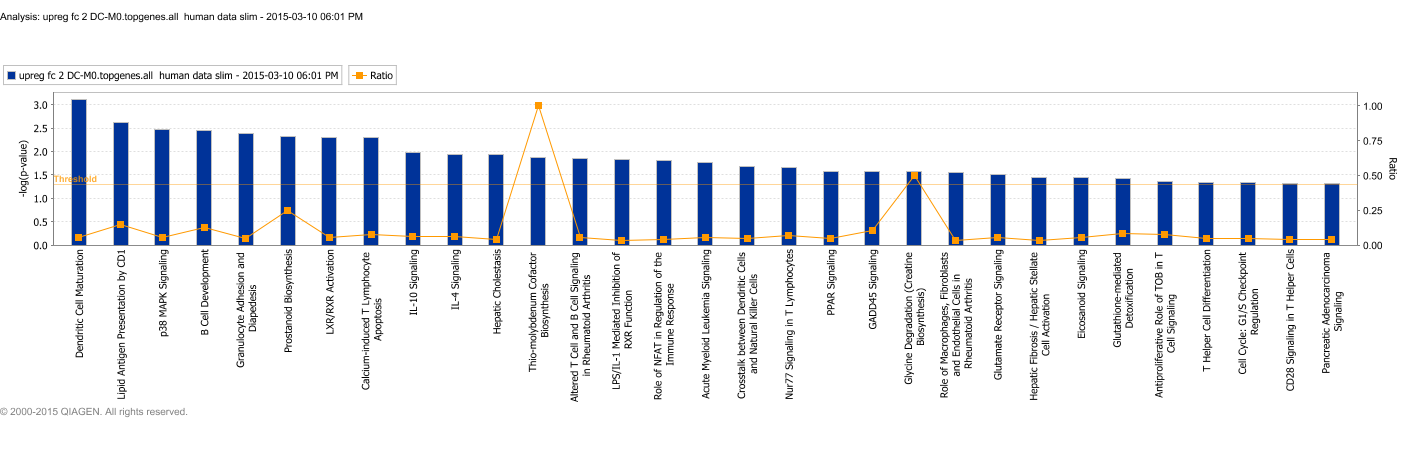
**
