# Supplementary figures and images for: Peritoneal macrophage heterogeneity is associated with different peritoneal dialysis outcomes
Source: Kidney Int. 2017 May;91(5):1088–103. doi: 10.1016/j.kint.2016.10.030 (PMC5402633; doi:10.1016/j.kint.2016.10.030)

**Figure S2.**

**
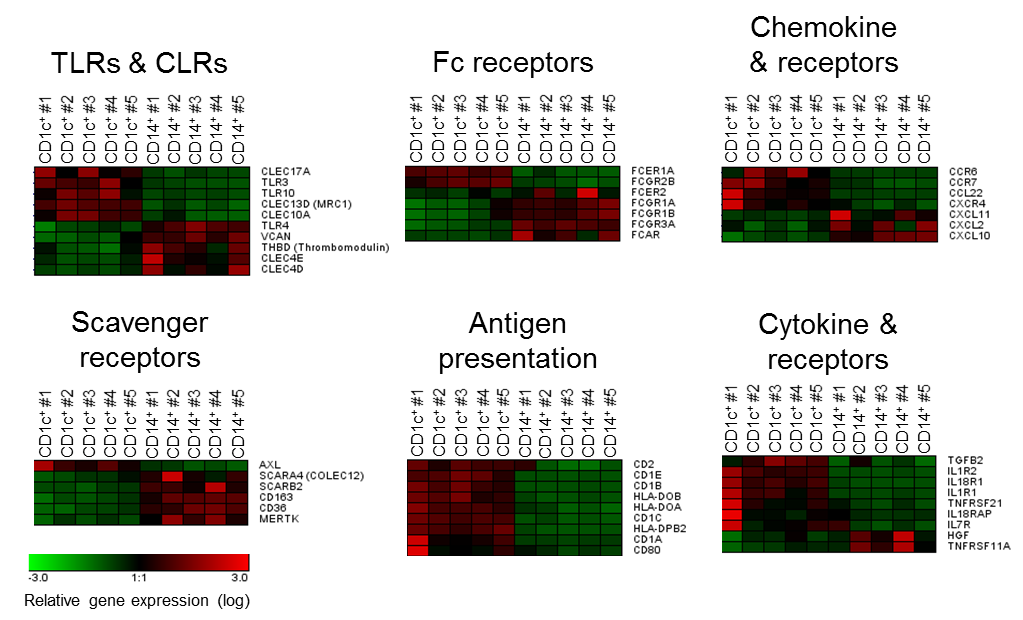
**

Supplement: Figure S2 — Peritoneal MØ and DC display distinct transcriptomic signatures. Heatmaps representing the relative expression of selective genes encoding key molecules involving major immune functions such as Toll-like receptors (TLR), c-type lectin receptors (CLR), scavenger receptors, Fc receptors, chemokine/receptors, cytokine/receptors, and molecules responsible for antigen presentation. Differentially expressed genes selected for analysis were based on fold change ≥2, either CD14+ cells versus CD1c+ cells or vice versa, then clustered on heat maps. [file mmc3.docx]

**Figure S3.**

**
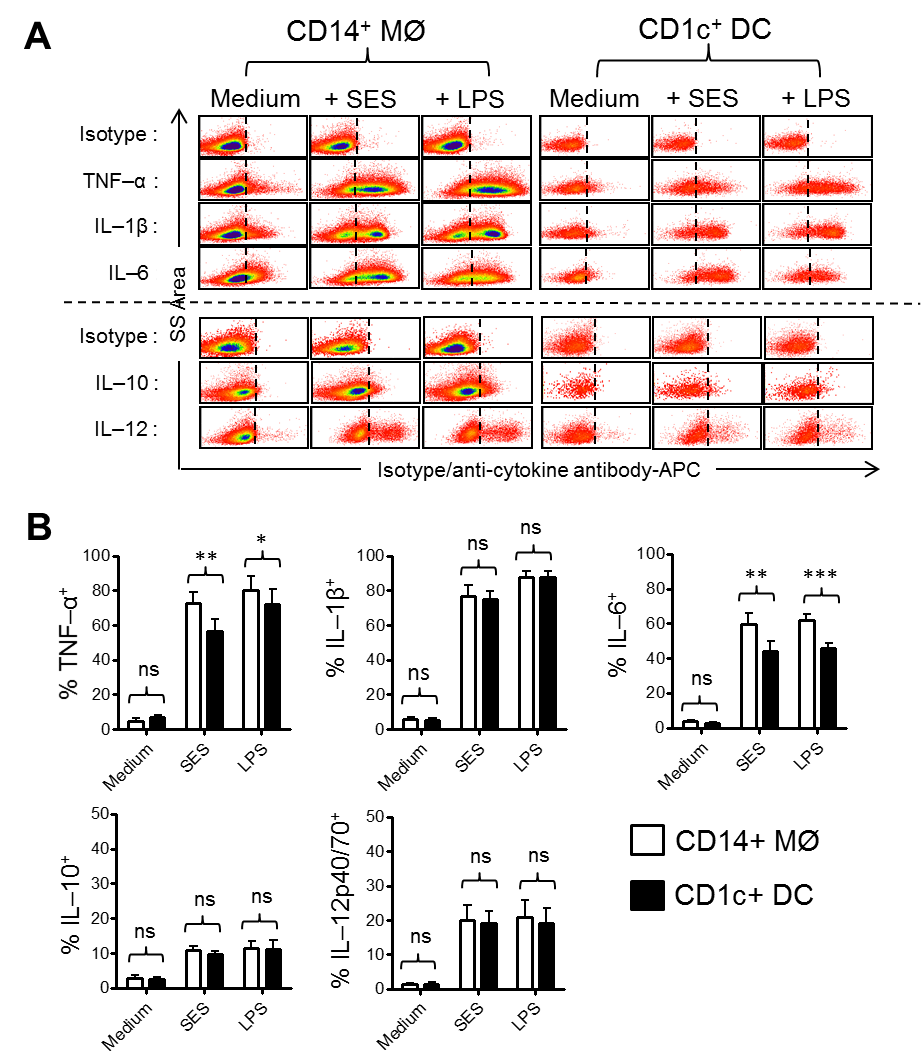
**

Supplement: Figure S3 — Cytokine production in response to ex vivo microbial product stimulation. (A) Representative density plots illustrating the flow-cytometric determination of intracellular cytokine production by peritoneal MØ/DC from stable PD patients, after ex vivo stimulation with S epidermidis supernatant (SES) (1:10 dilution) or lipopolysaccharide (LPS) for 4 hours (tumor necrosis factor [TNF]-α, IL-1β, IL-6) or 24 hours (IL-10), or 22 hours (IL-12p40/p70, in addition to 2 hours of interferon [IFN]-γ priming), in parallel with medium only as unstimulated control. Data are derived from 1 patient representative of 7 stable patients giving similar results. (B) Bar graphs showing the quantitative comparisons of the intracellular cytokine production between peritoneal CD14+ MØs and CD1c+ DCs. Cells were cultured in medium alone or stimulated ex vivo with SES or LPS as described previously. Positive intracellular cytokine production was determined by comparison with isotype controls. Data represent mean ± SEM and are derived from 7 stable PD patients. Statistical analysis was performed by paired t tests. [file mmc4.docx]

**Figure S4.**

**
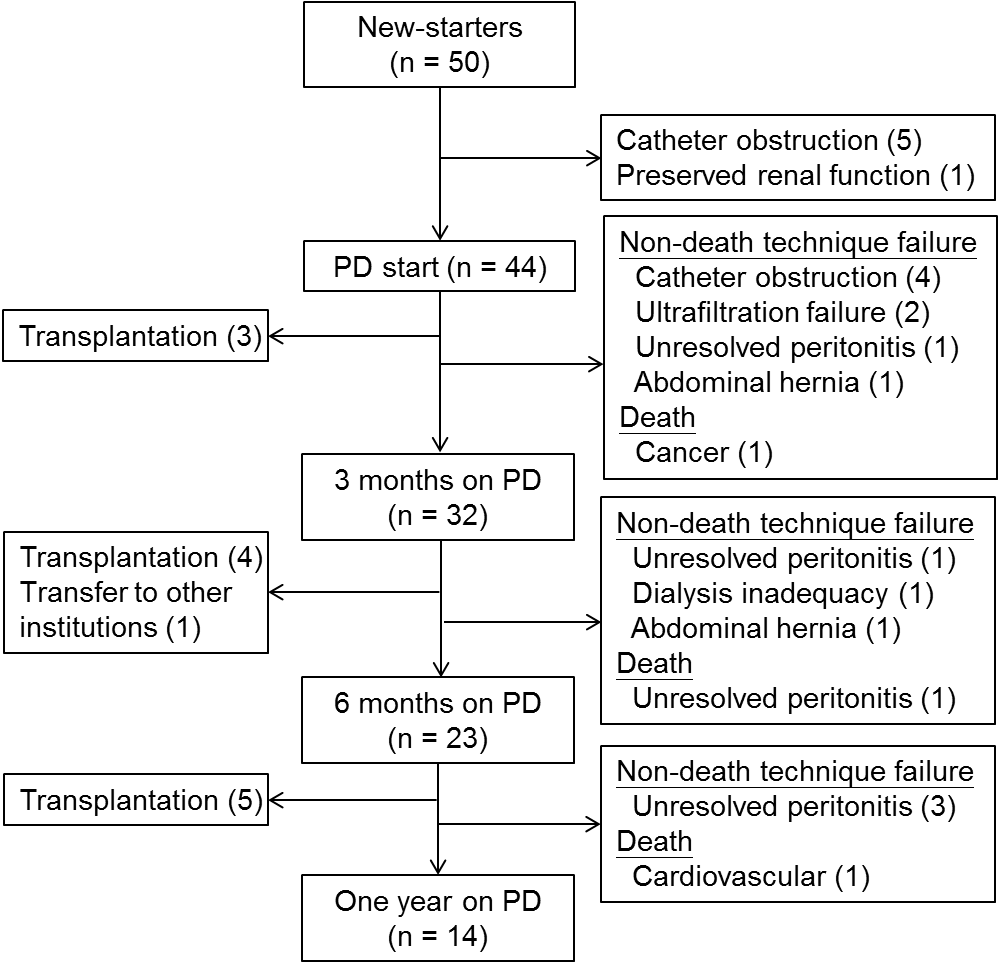
**

Supplement: Figure S4 — Flow diagram of longitudinal outcomes of new-starter PD patients during 1- year follow-up. A total of 50 new-starter PD patients was enrolled in this study. The reasons for dropout from this cohort during 1-year follow-up period included receiving kidney transplantation (n = 12), transferring to other institutions (n = 1), nondeath technique failure (n = 19), death (n = 3). The number in each set of parentheses denotes the number of patients under each defined category. [file mmc5.docx]

**Figure S5.**

**
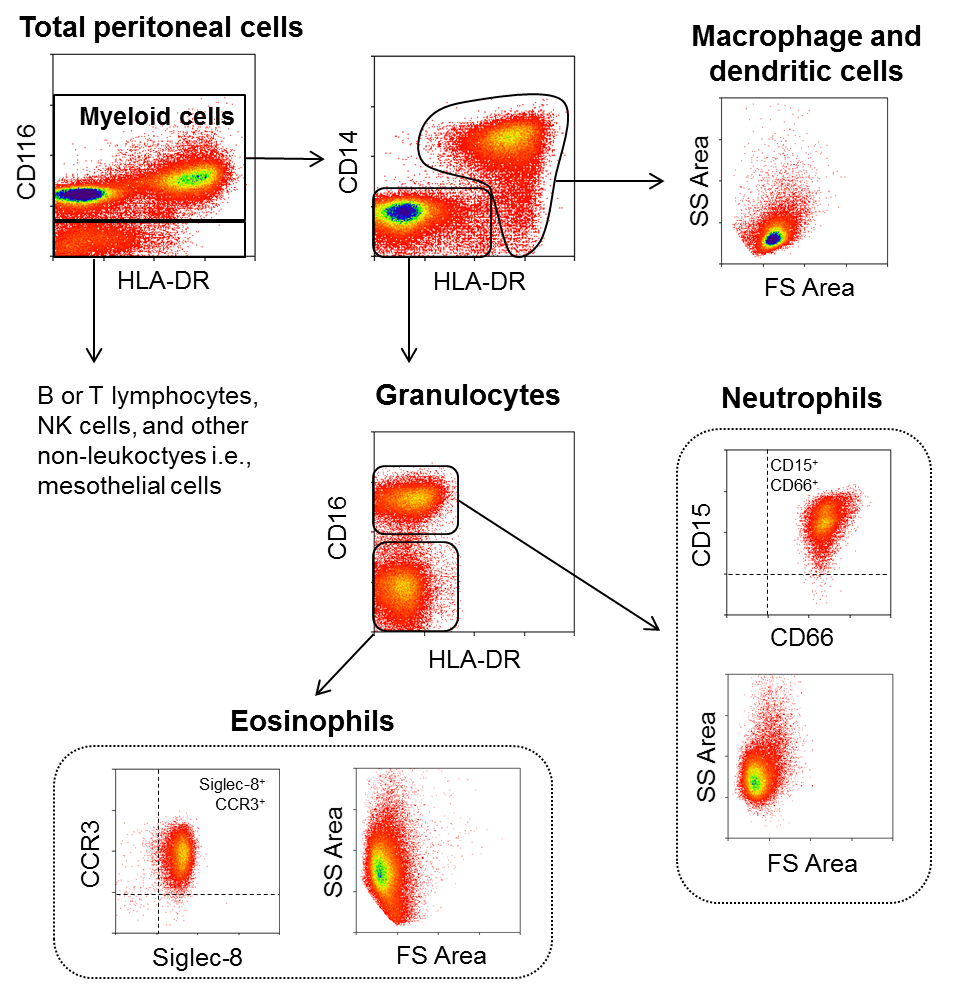
**

Supplement: Figure S5 — Flow-cytometric gating strategies to identify different peritoneal myeloid subsets from PD effluent fluids. Density plots show myeloid cells were pregated on CD116+ populations after exclusion of doublets, cellular debris, and dead cells. Within these cells, monocytic cells could be readily identified as HLA-DR+CD14+/low/–. Granulocytes were HLA-DR–CD14–. Other useful surface markers, such as CD16, CD15, CD66, CCR3, siglec-8, are applied for confirmation of neutrophils (CD16+CD15+CD66+) and eosinophils (CD16–CCR3+siglec-8+). Data represent the analysis of peritoneal effluent cells from the first flush bag of a new-starter PD patient. NK, natural killer. [file mmc6.docx]

**Figure S7.**

**
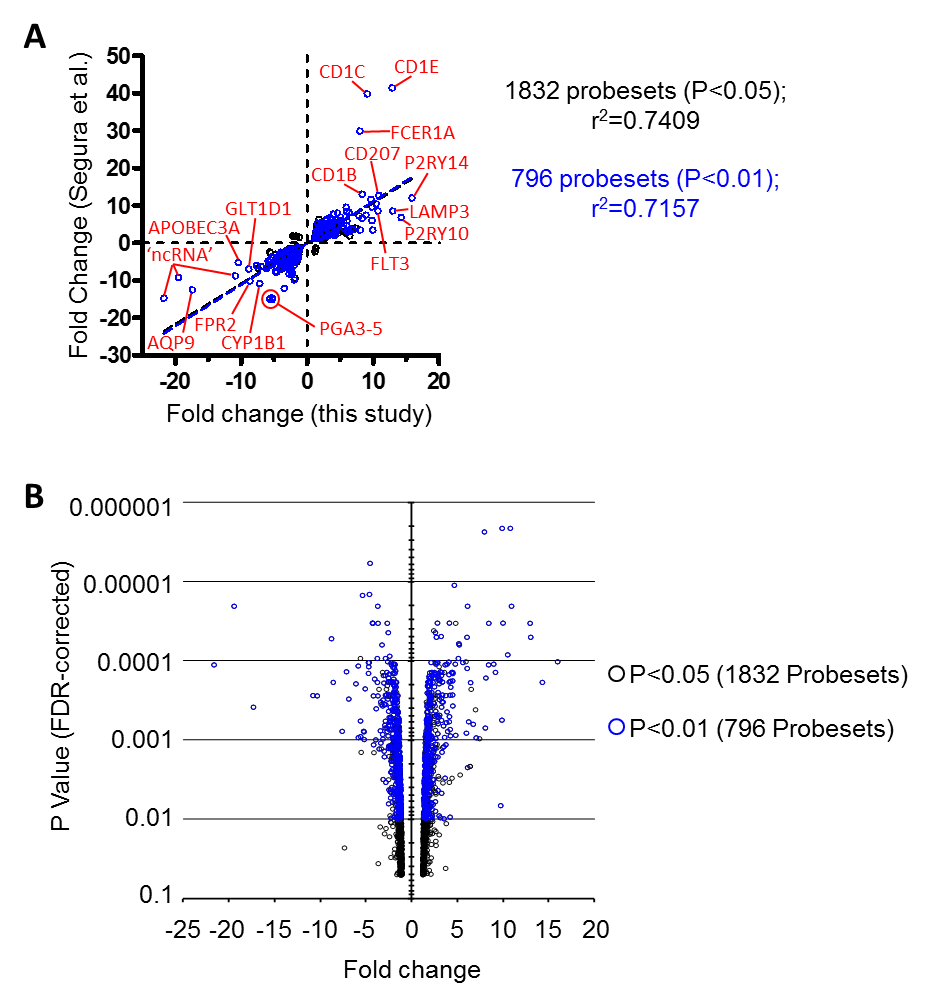
**

Supplement: Figure S7 — Comparison of 2 microarray datasets. The array data were generated (or downloaded) as described in the methods section. Our data were compared with that of Segura et al.41 (A) We compared the expression of the probesets that were significantly regulated in both our study and the Segura et al.41 dataset (between the 2 peritoneal subsets reported) (using 2 FDR-corrected P-value thresholds for regulation in the datasets as indicated). There was a high correlation between the expression patterns reported by Segura et al.41 and in our dataset, indicating likely overlap in the cellular identities. (B) The same data were also represented as a volcano plot to demonstrate fold change and P value in our dataset. [file mmc8.docx]
