# Supplementary material for: Peritoneal macrophage heterogeneity is associated with different peritoneal dialysis outcomes
Source: Kidney Int. 2017 May;91(5):1088–103. doi: 10.1016/j.kint.2016.10.030 (PMC5402633; doi:10.1016/j.kint.2016.10.030)
Supplement: Figure S6 — Changes in the number and the proportion of major peritoneal myeloid subsets during the first year of PD therapy. (A) Data showing the numbers of total peritoneal cells, neutrophils, eosinophils, and monocytic cells changed during the 1-year follow-up period. Data were representative of 171 PD samples from 50 patients at 6 time points (50 samples from 1st flush, 37 samples from 2nd flush, 39 samples from PD start, 20 samples from PD for 3 months, 15 samples from PD for 6 months, and 10 samples from PD for 1 year). Horizontal bar in each group represents the mean. (B) Graphs showing the numbers (left) and the proportion (right) of peritoneal neutrophils, eosinophils, and monocytic cells changed during the 1-year follow-up period. Data were derived from the same samples from (A). Error bars in each group denote the SEM. [file mmc7.docx]

**Figure 6.**

**A**

**B**

Under dialysis

Under dialysis
