# Supplementary material for: Peritoneal macrophage heterogeneity is associated with different peritoneal dialysis outcomes
Source: Kidney Int. 2017 May;91(5):1088–103. doi: 10.1016/j.kint.2016.10.030 (PMC5402633; doi:10.1016/j.kint.2016.10.030)
Supplement: Table S1 — Antibodies used in this study were purchased from BD Biosciences, Biolegend, eBioscience, or R & D Systems. Biotin-conjugated antibodies were detected with phycoerythrin (PE)-TexasRed-, allophycocyanin-, Peridinin-chlorophyll-protein complex [file mmc9.docx]

**Table S1.** Antibodies used in this study were purchased from BD Biosciences, Biolegend, eBioscience or R & D Systems. Biotin-conjugated antibodies were detected with phycoerythrin (PE)-TexasRed-, allophycocyanin-, Peridinin-chlorophyll-protein complex (PerCP)- or Alexa Fluor 405-conjugated streptavidin.

| **Antibody** | **Clone** |
| --- | --- |
| anti-CD1c-allophycocyanin/biotin | AD5-8E7 |
| anti-CD3-pacific blue | SK7 |
| anti-CD8-PE-Cy7 | SK3 |
| anti-CD11b-V450 | ICRF44 |
| anti-CD11c-V450 | B-ly6 |
| anti-CD14-ECD (PE-TexasRed) | RMO52 |
| anti-CD16-PE-Cy7 | 3GB |
| anti-CD19-PerCP | HIB19 |
| anti-CD36-allophycocyanin | 5-271 |
| anti-CD64 (FcγRI)-PE | 10.1 |
| Anti-CD66-PE | B1.1 |
| anti-CD80-PE | 2D10 |
| anti-CD86-PE-Cy5 | 2331 |
| anti-CD116-FITC | 4H1 |
| anti-CD163-PerCP/PE-Cy5.5/biotin | GHI/61 |
| anti-CD206-PE | 3.29B1.10 |
| anti-CD226-allophycocyanin | 11A8 |
| anti-CD301-allophycocyanin | H037G3 |
| anti-CCR2-allophycocyanin | 48607 |
| anti-CCR3-allophycocyanin | 5E8 |
| anti-CCR7-PE-Cy7 | 3D12 |
| anti-HLA-A2-allophycocyanin | BB7.2 |
| anti-HLA-DR-allophycocyanin-H7 | L243 |
| anti-FceR1-allophycocyanin | AER-37 |
| anti-Mer-allophycocyanin | 125518 |
| Anti-Siglec-8-PE | 7C9 |
| anti-IFN-γ-FITC | B27 |
| anti-TNF-α-allophycocyanin | 6401.1111 |
| anti-IL-1β-allophycocyanin | 8516 |
| Anti-IL-6-allophycocyanin | MQ2-13A5 |
| Anti-IL10-allophycocyanin | JES3-19F1 |
| Anti-IL-12(p40p70)-allophycocyanin | C8.6 |
